# Differential usage of DNA modifications in neurons, astrocytes, and microglia

**DOI:** 10.1186/s13072-023-00522-6

**Published:** 2023-11-13

**Authors:** Kyla B. Tooley, Ana J. Chucair-Elliott, Sarah R. Ocañas, Adeline H. Machalinski, Kevin D. Pham, Walker Hoolehan, Adam M. Kulpa, David R. Stanford, Willard M. Freeman

**Affiliations:** 1 Department of Physiology, University of Oklahoma Health Sciences Center, Oklahoma City, OK USA; 2 Genes & Human Disease Program, Oklahoma Medical Research Foundation, 825 NE 13th Street, Oklahoma City, OK 73104 USA; 3 Center for Biomedical Data Sciences, Oklahoma Medical Research Foundation, Oklahoma City, OK USA; 4 Department of Biochemistry, University of Oklahoma Health Sciences Center, Oklahoma City, OK USA; 5 Oklahoma City Veterans Affairs Medical Center, Oklahoma City, OK USA

**Keywords:** Brain, Epigenomics, Methylation, Hydroxymethylation, Regulatory elements, Genome regulation

## Abstract

**Background:**

Cellular identity is determined partly by cell type-specific epigenomic profiles that regulate gene expression. In neuroscience, there is a pressing need to isolate and characterize the epigenomes of specific CNS cell types in health and disease. In this study, we developed an in vivo tagging mouse model (Camk2a-NuTRAP) for paired isolation of neuronal DNA and RNA without cell sorting and then used this model to assess epigenomic regulation, DNA modifications in particular, of gene expression between neurons and glia.

**Results:**

After validating the cell-specificity of the Camk2a-NuTRAP model, we performed TRAP-RNA-Seq and INTACT-whole genome oxidative bisulfite sequencing (WGoxBS) to assess the neuronal translatome and epigenome in the hippocampus of young mice (4 months old). WGoxBS findings were validated with enzymatic methyl-Seq (EM-Seq) and nanopore sequencing. Comparing neuronal data to microglial and astrocytic data from NuTRAP models, microglia had the highest global mCG levels followed by astrocytes and then neurons, with the opposite pattern observed for hmCG and mCH. Differentially modified regions between cell types were predominantly found within gene bodies and distal intergenic regions, rather than proximal promoters. Across cell types there was a negative correlation between DNA modifications (mCG, mCH, hmCG) and gene expression at proximal promoters. In contrast, a negative correlation of gene body mCG and a positive relationship between distal promoter and gene body hmCG with gene expression was observed. Furthermore, we identified a neuron-specific inverse relationship between mCH and gene expression across promoter and gene body regions.

**Conclusions:**

Neurons, astrocytes, and microglia demonstrate different genome-wide levels of mCG, hmCG, and mCH that are reproducible across analytical methods. However, modification-gene expression relationships are conserved across cell types. Enrichment of differential modifications across cell types in gene bodies and distal regulatory elements, but not proximal promoters, highlights epigenomic patterning in these regions as potentially greater determinants of cell identity. These findings also demonstrate the importance of differentiating between mC and hmC in neuroepigenomic analyses, as up to 30% of what is conventionally interpreted as mCG can be hmCG, which often has a different relationship to gene expression than mCG.

**Supplementary Information:**

The online version contains supplementary material available at 10.1186/s13072-023-00522-6.

## Background

DNA methylation (mC) and hydroxymethylation (hmC) are stable modifications added to the 5 position of the cytosine ring in the CpG (CG) and non-CpG (CH) contexts, each (mCG, hmCG, and mCH) having distinct roles in genome regulation and gene expression in the central nervous system (CNS) [1]. The presence of hmCH is debated, and its potential role in genome regulation has yet to be elucidated [2–4]. DNA modification patterns modulate CNS cell differentiation and specialization [5–8], with deposition and removal occurring at different points of neurodevelopment [2]. While mCG has been extensively studied, there is increasing interest in investigating hmCG and mCH in neuroscience research due to their higher abundance in the brain compared to other tissues [9–11] and their potential involvement in neurological disease [12–16]. Notably, the deposition of mCH coincides with increased synaptic density and a positive association between gene body hmCG and gene expression suggests potential functional roles of DNA modifications in both neurodevelopment and the adult brain [2, 17].

The complete role of DNA modifications in regulating gene expression is still being determined, but recent advances have revealed that different modifications have distinct relationships to gene expression that can vary by genomic context. Methylation exerts a well-established repressive function on gene expression when deposited in the proximal promoter region [18], with the caveat that most reported ‘methylation’ data in the field is derived from bisulfite methods that cannot differentiate between mCG and hmCG, so what is reported as mCG is actually total modifications. Methyl-binding proteins, such as MeCP2, recognize and bind to methylated DNA, further impeding transcription and reinforcing the repressive effect of mCG [19]. This repressive function can have long-lasting effects, as mCG plays a crucial role in the long-term repression of repetitive elements and X-chromosome inactivation within the CNS [10, 11]. Within gene bodies, mCG has been described exhibiting both a negative [3, 20] and positive [21] relation to gene expression, leaving the functional relationship of this modification to gene expression somewhat ambiguous. In contrast, hmCG is positively correlated with gene expression and is enriched at tissue-specific genes and transcription factor binding sites [22, 23]. In postmitotic neurons, hmCG is primarily located in the gene body of expressed genes, and has been interpreted as “functional demethylation” of these regions, serving to decrease binding affinity of MeCP2 and promote gene expression [24, 25]. Furthermore, it is hypothesized that hmCG may be required for development of the complex morphology and synaptic connections of long-range postmitotic neurons [26, 27].

Methylation in CH contexts (C followed by C, A, or T) was not previously considered a prominent site of cytosine methylation. However, neurons have the highest proportion of mCH observed in the body [2]. mCH is depleted within highly expressed genes and their regulatory elements, instead potentially serving to fine tune the cell type-specific expression of lowly expressed neuronal genes [6, 28]. Additionally, mCH accumulation during development parallels synaptogenesis, indicating that mCH is likely important in regulating the formation and maintenance of synaptic connections [29].

Although neuro-epigenomics research has advanced considerably, the field lacks comprehensive cell type-specific maps of the relationships between DNA modifications (mCG, hmCG, mCH) and gene expression in health and disease. This is largely due to the challenges of isolating specific CNS cell populations and the aforementioned reliance on bisulfite sequencing methods that cannot differentiate between mC and hmC. Additionally, many studies have not examined CH modifications due to not collecting this data (as is the case with methylation arrays) or the difficulties of the bioinformatic analyses needed to extract this data from sequencing studies. To address the technical and knowledge gaps in the field we combine the Nuclear Tagging and Translating Ribosome Affinity Purification (NuTRAP) mouse line [30–32] with a well-established neuronal-specific inducible cre-recombinase system (Camk2a-cre/ERT2 [33–36]) to perform a paired translatomic and epigenomic analysis of excitatory glutamatergic pyramidal neurons in the hippocampus [37–40]. To gain a broader perspective, we compare our neuronal findings to astrocytic and microglial data [41]. This comparative analysis reveals cell-type specific usage of DNA modifications and their associations with mRNA expression across three CNS cell types. Studies described here: (1) validate the Camk2a-NuTRAP model, (2) compare DNA modification usage across three CNS cell types, and (3) assess the relationship between DNA modifications and mRNA levels in the three CNS cell types, providing insight into the regulatory mechanisms governing gene expression. By undertaking these investigations, we hope to advance the understanding of the role of DNA modifications in gene regulation across different CNS cell types, paving the way for future discoveries in neuro-epigenomics.

## Results

### Immunohistochemical validation of the Camk2a-NuTRAP mouse brain

To avoid interference with neurodevelopmental processes, we performed tamoxifen (Tam) induction of Camk2a-NuTRAP in mature adult mice at 3 months of age (3mo). This timing was chosen to circumvent deficits in spatial learning, contextual fear memory, and presynaptic structure that can arise after perturbing Camk2a expression during early neurodevelopment [42, 43]. Brains were collected one month following Tam induction and sectioned sagittally for immunohistochemical analysis.

**Fig. 1:**
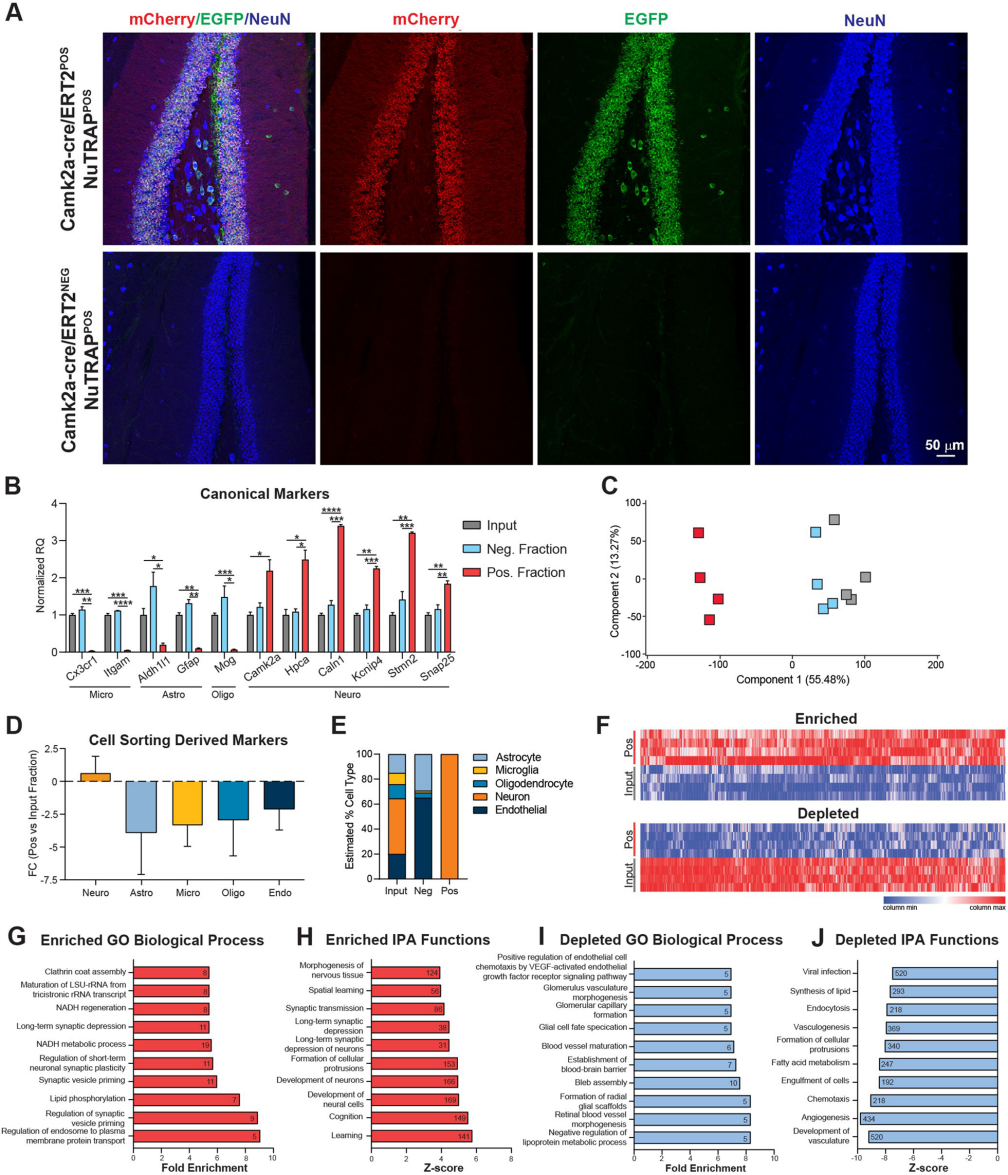
Validation of neuronal translatome enrichment in TRAP-RNA from Camk2a-NuTRAP mouse hippocampus. **A** Imaging of the hippocampal dentate gyrus demonstrated EGFP and mCherry co-expression in NeuN + cells. **B** TRAP-isolated hippocampal RNA from input, negative, and positive fractions were assessed by qPCR for enrichment and depletion of canonical marker genes for microglia, astrocytes, oligodendrocytes, and neurons. Mean relative gene expression ± SEM scaled to input for each gene. *p < 0.05, **p < 0.01, ***p < 0.001, ****p < 0.0001 by RM one-way ANOVA with Tukey’s multiple comparison test across fractions (*n* = 4/group). **C** RNA-seq was performed for all fractions (*n* = 4/group). Principal component analysis shows separation of the positive from input and negative fraction samples in the first component. **D** Cell-type marker gene lists were examined for fold change (Positive/Input) enrichment or depletion shows enrichment of neuronal markers and depletion of other cell-type markers in the positive fraction. **E** CIBERSORTx calculation of cell type composition of each fraction. The positive fraction is estimated to contain 100% neurons. **F** Genes with significant enrichment (2111) or depletion (2897) in the positive compared to input fraction were identified (FC >|1.25|, p < 0.05, Benjamini Hochberg multiple testing corrections). **G**–**J** Gene Ontology enrichment analysis and Ingenuity Pathway Analysis performed on significantly enriched or depleted genes (Positive/Input fraction) identified in **E**

Immunostaining of Camk2a-NuTRAP (Camk2a-cre/ERT2^+^; NuTRAP^+^) brains showed EGFP and mCherry colocalization in cells expressing the pan-neuronal marker NeuN. No EGFP or mCherry expression was seen in Camk2a-cre negative counterparts (Fig. 1A) and minimal expression was observed in a portion of NeuN^+^ cells of Camk2a-NuTRAP (-Tam) brains, which is consistent with previous reports (Additional file 10: Figure S1A-B) [36, 44]. Camk2a-NuTRAP (+ Tam) brains show no expression of EGFP in microglial (CD11b^+^), endothelial (CD31^+^), or astrocytic (GFAP^+^) cells (Additional file 10: Figure S1C–E). Collectively, these findings indicate a robust neuronal-specific and tamoxifen-dependent induction of the NuTRAP allele. This validation ensures that the experimental manipulations specifically target neuronal cells while minimizing any confounding effects on other cell types in the brain.

### Validation of neuronal translatome enrichment from TRAP-isolated RNA

Translating RNA was isolated from the hippocampus of Camk2a-NuTRAP mice via the TRAP method (Translating Ribosome Affinity Purification) [41]. Subsequent RT-qPCR of RNA from the input, negative, and positive TRAP fractions showed a significant enrichment of neuronal marker genes (*Camk2a*, *Hpca*, *Caln1*, *Kcnip4*, *Stmn2*, and *Snap25*) in the positive fraction compared to input and negative fraction. Conversely, there was a depletion of microglial (*Cx3cr1* and *Itgam*), astrocytic (*Aldhl1l* and *Gfap*), and oligodendrocytic (*Mog*) marker genes in the positive fraction as compared to the input and negative fraction (Fig. 1B; Additional file 1B).

To further characterize the neuronal translatomic profile, TRAP-isolated RNA was subjected to RNA-Seq. Principal Component Analysis (PCA) of all expressed genes revealed clear separation of the positive fraction from input and negative fraction in the first component (Fig. 1C). To validate the TRAP-enrichment of neuronal genes and depletion of other cell type-specific genes, we used marker lists generated from previous cell sorting studies [41] (Additional file 2). Enrichment of neuronal genes and depletion of astrocytic, microglial, oligodendrocytic, and endothelial genes was evident in the positive fraction as compared to input (Fig. 1D; Additional file 3A). Notably, there was a high fold depletion of markers for minority cell types like glia and smaller fold-change enrichment for neurons, which make up the majority of the input.

To estimate the cell type composition of the input, negative and positive fractions, we employed CIBERSORTx [45] using established cell type marker lists [41]. This analysis revealed that the input contained the expected variety of cell types at the expected proportions (astrocytes, microglia, neurons, oligodendrocytes, and endothelial cells). The negative fraction demonstrated a depletion of neuronal cells, whereas the positive fraction was estimated to be entirely represented by neurons (~ 100%) (Fig. 1E; Additional file 3B).

Ingenuity Pathway Analysis and Gene Ontology analysis of the significantly enriched and depleted genes in the positive fraction vs input (Fig. 1F; Additional file 3C,D) revealed enriched genes regulating excitatory neuronal biological processes and functions such as those involved in synaptic structure, maintenance and plasticity (Fig. 1G, H; Additional file 3E,F). On the other hand, depleted genes were involved in lipid metabolism, immune response, and vascular formation and maintenance, indicating a depletion of genes involved in non-neuronal pathways (Fig. 1I, J**; **Additional file 3G, H). Moreover, the positive fraction enrichment of genes involved in spatial learning, a major function of hippocampal neurons, further demonstrated the specificity and relevance of the model in capturing neuronal-specific transcripts [46, 47]. These findings provide valuable insights into the enriched and depleted gene sets within the neuronal translatome, shedding light on the functional processes and pathways associated with neuronal identity and function of the hippocampus.

### Validation of neuronal gDNA isolation from INTACT Whole Genome Bisulfite Sequencing

**Fig. 2:**
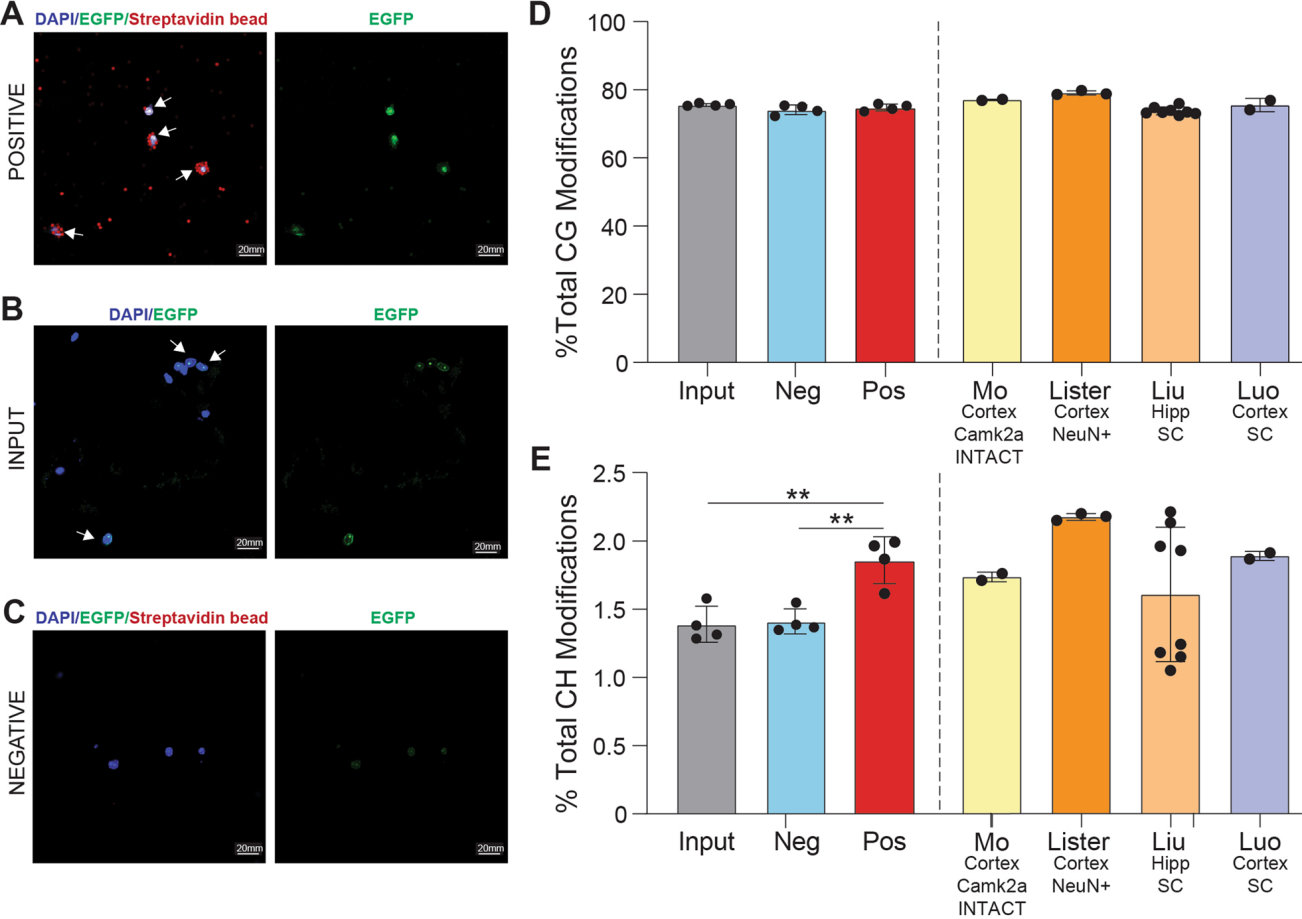
Validation of neuronal genome enrichment in Camk2a-NuTRAP mouse hippocampus by INTACT-BS seq. **A**–**C** Confocal fluorescent microscopy images from positive, input, and negative INTACT nuclei isolations. **D** INTACT-isolated gDNA from the hippocampus of Camk2a-NuTRAP mice was bisulfite converted and whole genome levels of CG modifications measured for input, negative, and positive fractions. CG modifications from previously published neuronal methylation studies utilizing various brain regions (hippocampus and cortex) and isolation techniques (Camk2a INTACT, NeuN^+^ sorting, and single cell) were compared to Camk2a-NuTRAP CG modifications. **E** Whole genome CH modifications were measured for input, negative, and positive fractions. CH modifications from the same neuronal methylation studies from **D** were compared to Camk2a-NuTRAP CH modifications (**p < 0.01 by one-way ANOVA with Tukey’s multiple testing correction)

To ensure the purity of the positive fraction obtained through INTACT isolation (Isolation of Nuclei in TAgged in specific Cell Types), expression of EGFP within the nucleus [32] was assessed by confocal microscopy. EGFP-positive nuclei surrounded by streptavidin beads were observed in the positive fraction (Fig. 2A). In contrast, the input showed a mixture of EGFP-positive and EGFP-negative nuclei (Fig. 2B), while the negative fraction exhibited no EGFP expression (Fig. 2C).

To assess DNA modifications in the positive fraction, whole genome oxidative bisulfite sequencing (WGoxBS) was performed on INTACT-isolated gDNA from the input, negative, and positive fractions to measure mC and hmC in the CG and CH contexts. First, the bisulfite-only arm, which detects a combined signal of mC and hmC (total modifications), was compared to previously published neuronal bisulfite sequencing data. Total CG and CH modification levels from the positive fraction were similar to previously published neuronal bisulfite sequencing modification studies [2, 48–50] **(**Fig. 2D–E**)**. These findings provide evidence for the isolation of neuronal-specific genomic DNA, supporting the validity of the INTACT isolation method and the subsequent analysis of DNA modifications in the positive fraction.

### Neuronal epigenome analysis using whole genome oxidative bisulfite sequencing

**Fig. 3:**
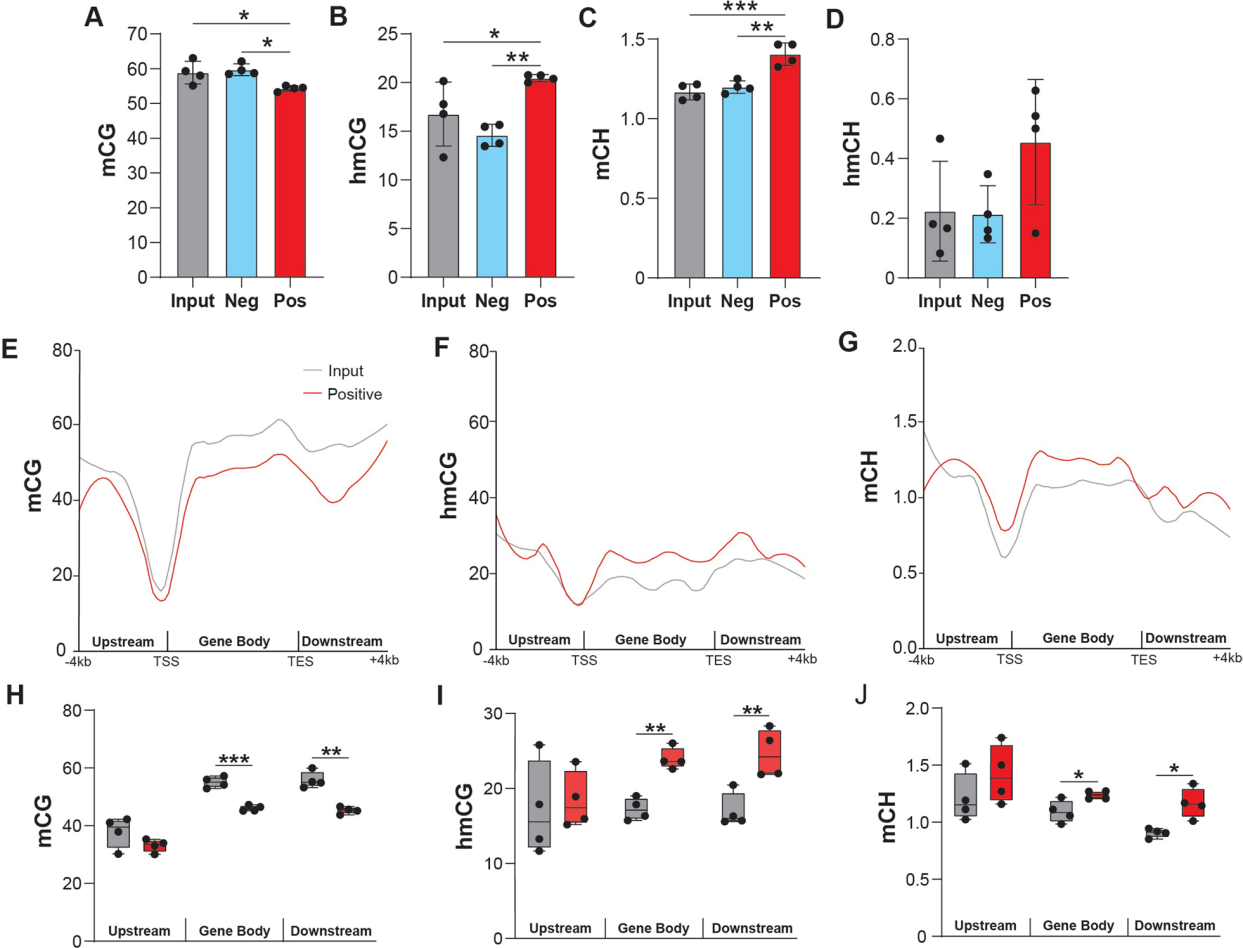
Profile of hippocampal neuronal DNA modifications by whole genome oxBS-seq. INTACT hippocampal gDNA from input, negative and positive fractions was taken for whole genome bisulfite and oxidative bisulfite sequencing. **A–D** Total genomic levels of mCG, hmCG, mCH, and hmCH (*n* = 4/group; one-way ANOVA with Tukey’s multiple comparisons test, *p < 0.05, **p < 0.01, ***p < 0.001). Levels of mCG were lower and hmCG and mCH were higher in the positive fraction. **E**–**G** mCG, hmCG, and mCH averaged over 200 nucleotide bins from 4 kb upstream, within the gene body, and 4 kb downstream of neuronal marker genes in the positive fraction and input. **H**–**J** Average mCG, hmCG, and mCH for positive fraction and input 4 kb upstream of the TSS, within the gene body, and 4 kb downstream of the TES of neuronal genes revealed lower mCG and higher hmCG and mCH in gene bodies and downstream (*n* = 4/group; paired two-tailed t-test between input and positive fraction, *p < 0.05, **p < 0.01, ***p < 0.001)

To distinguish between mC and hmC, the oxidative bisulfite sequencing (oxBS-Seq, mC only) arm was subtracted from the bisulfite sequencing (BS-Seq, mC + hmC) arm for INTACT-isolated DNA from the input, negative, and positive fractions (Additional file 10: Figure S2A). Conversion efficiency, measured by spike-in controls, was close to 100% with no significant variance between samples or groups (Additional file 10: Figure S2B–C**, **Additional file 4). Comparing the different fractions, the positive fraction exhibited significantly lower mCG and higher levels of hmCG and mCH when compared to input and negative fractions (Fig. 3A–C). Non-CG hydroxymethylation (hmCH) was detected at low levels near background (< 1%) and was not significantly different between fractions (Fig. 3D).

Deeper sequencing (1-2X) was performed on the input and positive fraction **(**Additional file 5**)**, with the knowledge that only 0.001X coverage is required for obtaining accurate whole genome and repeat element modification levels within 1% [51]. Distribution of DNA modifications (mCG, hmCG, and mCH) was also mapped across genic regions (Promoter, Gene Body, Downstream) of neuronal marker genes (Additional file 2). In the positive fraction, the intragenic/gene body and downstream regions of neuronal marker genes showed significantly lower mCG levels and significantly higher mCH and hmCG levels compared to input (Fig. 3E–J). Neuronal marker genes had lower mCG, hmCG, and mCH at the TSS compared to all genes (Additional file 10: Figure S3), with lower mCG observed across the gene body of neuronal genes compared to all genes (Additional file 10: Figure S3A).

### Comparison of DNA modifications across three CNS cell types

We previously validated the use of the NuTRAP construct in two mouse lines for isolation of gDNA and RNA from astrocytes and microglia [41]. To compare the DNA modification profiles between neurons, astrocytes and microglia, previously published WGoxBS sequencing data from the positive fractions of Aldh1l1-NuTRAP and Cx3cr1-NuTRAP (GSE140271) were compared to Camk2a-NuTRAP (present study, GSE228044).

**Fig. 4:**
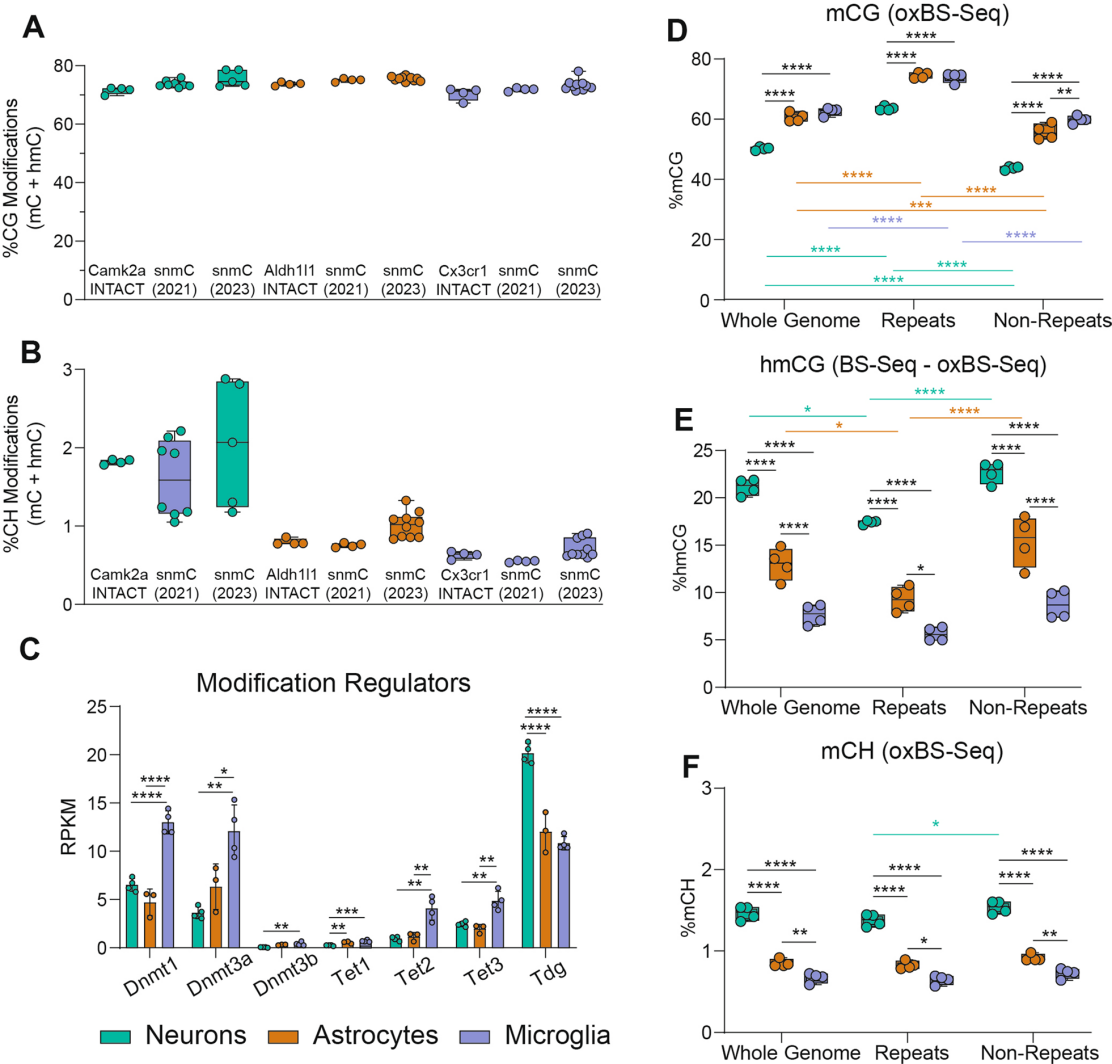
Comparison of DNA modifications across three CNS cell types. Whole genome total CG modifications **A** and CH modifications **B** from INTACT-isolated gDNA from neurons (hippocampus), astrocytes (half brain), and microglia (half brain) were compared to two single nuclei methylome studies [49, 52]. **C** TRAP RNA-seq expression of DNA modification regulators in neurons, astrocytes, and microglia (*n* = 3–6/group; one-way ANOVA with Tukey’s multiple comparisons test, *p < 0.05, **p < 0.01, ***p < 0.001, ****p < 0.0001) data presented at reads per kilobase mapped. **D–F** Whole genome, repetitive element, and non-repetitive element mCG, hmCG, and mCH levels for neurons, astrocytes, and microglia. (*n* = 4/group; two-way ANOVA with Tukey’s multiple comparisons test, *p < 0.05, **p < 0.01, ***p < 0.001, ****p < 0.0001)

Whole genome total CG modifications (by BS-Seq) were consistent across cell types, as well as between INTACT WGoxBS and single nuclei methylome studies (snmC) [49, 52] (Fig. 4A). Alternatively, whole genome total CH modifications were consistent within cell types, with neuronal levels being nearly twice as high as astrocytes or microglia (Fig. 4B). When distinguishing between mCG and hmCG, neurons exhibited higher hmCG levels and lower mCG levels compared to astrocytes and microglia (Fig. 4D–E). To better understand the origin of observed DNA modification differences between cell types, we assessed the cell type-specific expression of modification regulators [DNA methyltransferases (DNMTs), Ten–eleven translocases (TETs), and thymine DNA glycosylase (TDG)]. Microglia had significantly higher DNMT (*Dnmt1*, *Dnmt3a*, and *Dnmt3b*) expression than astrocytes and microglia, aligning with microglia having the highest levels of mCG among the three cell types (Fig. 4C). Surprisingly, despite having the lowest hmCG of the three cell types assessed, microglia also express TETs (*Tet1*, *Tet2*, and *Tet3*) at a significantly higher level compared to neurons and astrocytes (Fig. 4C). TET2 has been previously shown to regulate the microglial type I interferon-mediated inflammatory response upon LPS administration [53], pointing to a potentially dynamic role for microglial hydroxymethylation in modulating cell phenotype.

On the other hand, TDG, which mediates base-excision repair in active demethylation and single-strand break repair, was most highly expressed in neurons (Fig. 4C). As such, the methylation and demethylation cycle may serve as a source of site-specific neuronal single-strand breaks that have been previously observed within enhancer elements [54]. DNMTs, TETs, and TDG were also examined in public single cell gene expression repositories (Tabula Muris [55], Allen Brain [56], and Aging Mouse Brain atlases (young timepoint only) [57]), but due to comparative insensitivity of scRNA-Seq to TRAP-Seq, many of these genes were at the limit of detection and did not demonstrate clear patterns of differences between cell types (Additional file 10: Figure S4).

Repetitive elements comprise over 50% of the genome and are thought to play an important role in neuronal differentiation and maturation [58, 59]. To determine the genomic localization of the observed cellular DNA modification differences, the levels of DNA modifications in whole genome, repeat elements, and non-repeat elements were assessed in neurons, astrocytes, and microglia. mCG levels were lower in neurons across the whole genome, repeat, and non-repeat elements compared to astrocytes and microglia (Fig. 4D). Conversely, hmCG levels were significantly higher in neurons across repeat and non-repeat elements than astrocytes and microglia, with microglia exhibiting the lowest hmCG levels among the three cell types (Fig. 4E). In the CH context, a similar pattern was observed across the genome and when split between repeat and non-repeat elements (Fig. 4F).

Furthermore, when examining the split of whole genome levels into repeat and non-repeat elements, it was observed that repetitive elements had significantly higher mCG levels, while non-repetitive elements had significantly lower mCG levels compared to whole genome levels (Fig. 4D). Conversely, there was significantly lower hmCG levels in repetitive elements compared to the whole genome levels in neurons and astrocytes, with no difference between non-repetitive elements and whole genome levels for neurons, astrocytes or microglia (Fig. 4E).

In general, CG modification levels between cell types of repetitive and non-repetitive elements followed the pattern observed in whole genome levels. On the other hand, mCH levels were consistently the highest in neurons followed by astrocytes and then microglia, regardless of the genomic context across whole genome, non-repetitive, or repetitive elements (Fig. 4F). Unlike CG modifications, mCH levels were observed to be nearly identical across repetitive and non-repetitive elements. These findings provide insights into the cell type-specific distribution of DNA modifications across different genomic regions, including the impact of repeat elements, and highlight the distinct epigenetic landscapes in neurons, astrocytes, and microglia.

**Fig. 5:**
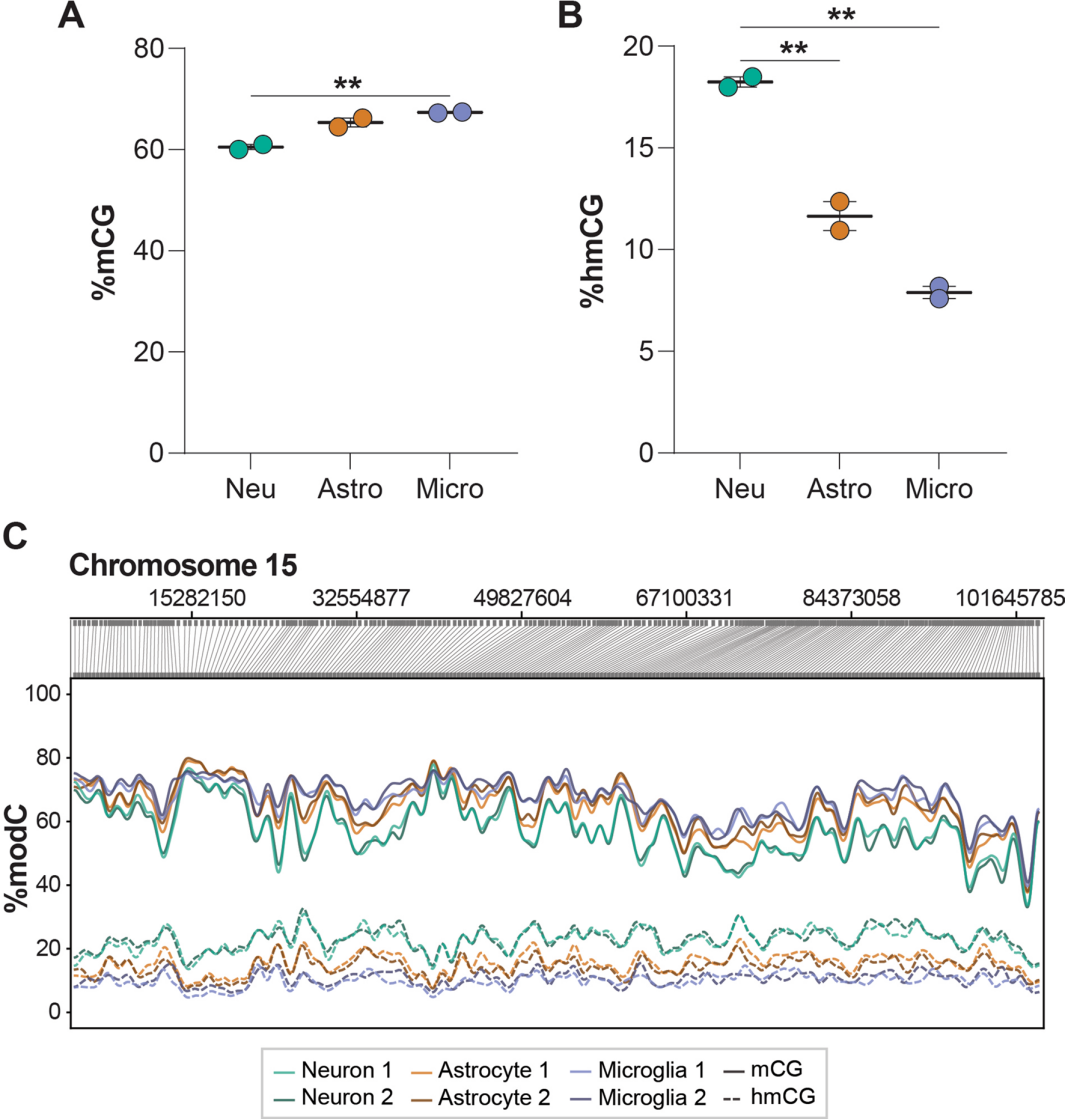
Native detection of DNA modifications with nanopore long-read sequencing. Nanopore long-read sequencing was performed on INTACT-isolated high molecular weight gDNA from neurons, astrocytes, and microglia (*n* = 2/group). Native mCG and hmCG calling was performed to obtain total whole genome %mCG **A** and %hmCG **B**. %modC (%mCG or %hmCG) was plotted across chromosome 15 **C** to demonstrate modification differences between cell types and reproducibility across biological replicates. Modification values were smoothed in CpG-only coordinate space (One-way ANOVA with Tukey’s multiple comparisons test, *p < 0.05, **p < 0.01)

To further validate the whole genome CG methylation and hydroxymethylation values obtained by WGoxBS, long-read nanopore sequencing and native CG methylation and hydroxymethylation calling was performed on separate INTACT-isolated high molecular weight gDNA from neurons, astrocytes, and microglia (*n* = 2/group; PRJNA1026932). As was observed from WGoxBS, neurons had lower mCG (Fig. 5A) and higher hmCG (Fig. 5B) levels compared to astrocytes and microglia. The absolute values obtained were also highly consistent with the WGoxBS data and between biological replicates (Fig. 5C). The pattern of modification levels between cell types is observed across entire chromosomes, as is represented by Chromosome 15 (Fig. 5C). Overall, the same pattern of cell type differences in mCG and hmCG were observed with both WGoxBS and nanopore sequencing, offering further validation for the DNA modification levels reported above. A table of average whole genome mCG and hmCG measured with WGoxBS, conversion corrected oxBS, and Nanopore can be found in Additional file 10: Table S1.

**Fig. 6:**
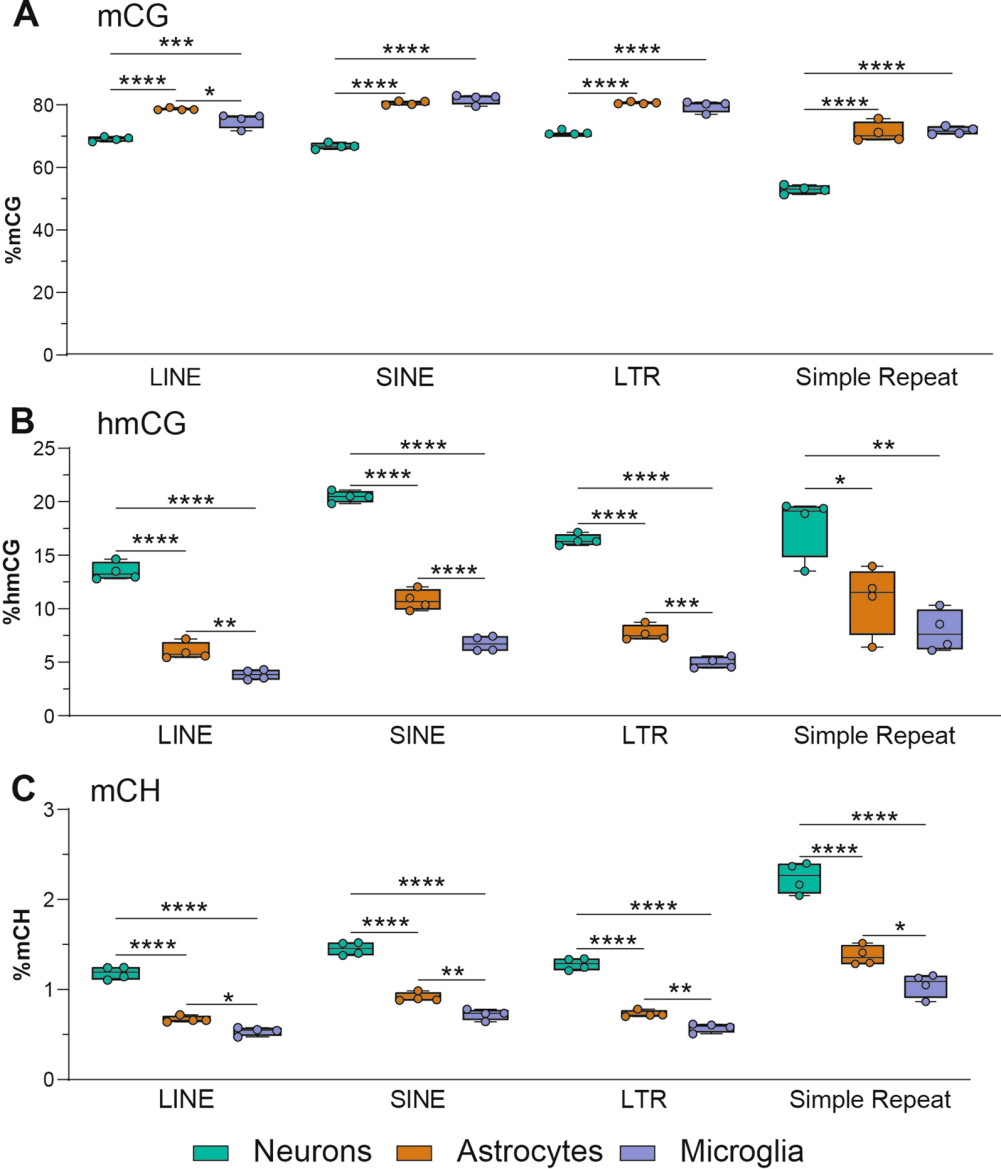
Repeat element DNA modifications in the CNS. mCG **A**, hmCG **B**, and mCH **C** levels of LINE, SINE, LTR, and Simple Repeat elements for neurons, astrocytes, and microglia. (*n* = 4/group; one-way ANOVA with Tukey’s multiple comparisons test, *p < 0.05, **p < 0.01, ***p < 0.001, ****p < 0.0001)

Repetitive elements are a known source of somatic mosaicism in the brain, and their aberrant activity (particularly LINE1) is implicated in several neurological and neurodegenerative diseases [60]. We next compared the DNA modification levels between neurons, astrocytes, and microglia in specific repeat elements: long interspersed nuclear elements (LINEs), short interspersed nuclear elements (SINEs), long terminal repeats (LTRs), and simple repeats. Neurons had lower mCG levels compared to astrocytes and microglia in all analyzed repeat elements (LINEs, SINEs, LTRs, and simple repeats) (Fig. 6A). Consistent with the whole genome levels, neurons had the highest level of hmCG and mCH within LINEs, SINEs, LTRs, and simple repeats, whereas microglia had the lowest levels of these modifications (Fig. 6B–C). Compared to whole genome levels, mCG levels were higher within repetitive elements (LINEs, SINEs, LTRs, and simple repeats), whereas repetitive hmCG and mCH levels were lower than whole genome. The only exception to this was mCH levels within simple repeats [2.51% (neuron), 1.54% (astrocyte), 1.17% (microglia)], which were higher than whole genome levels [1.46% (neuron), 0.85% (astrocyte), 0.67% (microglia)] (Fig. 6A–C). Additionally, simple repeats use more mCH and less mCG compared to other specific repeat elements analyzed (Fig. 6A, C). The mCG and hmCG levels in LINEs, SINEs, and LTRs were also assessed from nanopore long-read sequencing, and were overall consistent with WGoxBS (Additional file 10: Figure S5). Generally, the modification patterns between specific repeat elements followed the patterns observed at the whole genome level. However, there were differences in the absolute levels of DNA modifications depending on the specific repeat element analyzed.

### Differential DNA modifications are enriched at cell-type specific transcription factor motifs

**Fig. 7:**
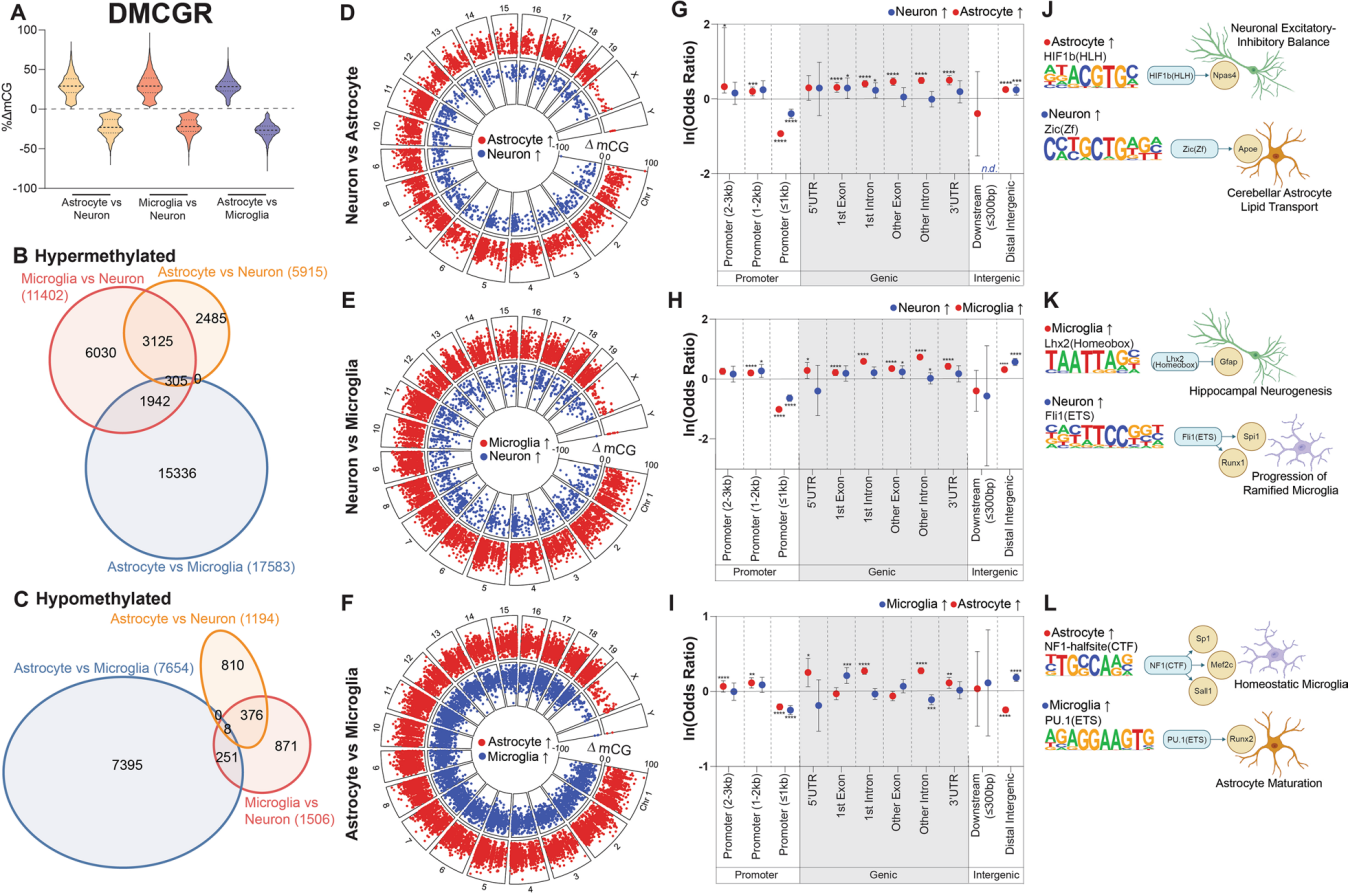
Differentially methylated CG regions. Differentially methylated CG regions (DMCGRs) were determined between cell types. Distribution of mCG differences **A** was plotted, along with overlap of hyper- **B** and hypo- **C** DMCGRs between the three comparisons. Genomic distribution and magnitude of DMCGRs for astrocytes vs neurons **D**, microglia vs neurons **E**, and astrocytes vs microglia **F**. Relative over- and under-representation in genic features for astrocytes vs neurons **G**, microglia vs neurons **H**, and astrocytes vs microglia **I**. Top enriched transcription factor binding motifs for astrocytes vs neurons **J**, microglia vs neurons **K**, and astrocytes vs microglia **L**. Genic regions containing no DMCGRs were notated as “*n.d.*” (Woolf logit method for 95% confidence intervals, Fisher’s exact test for two-sided p-values; *p < 0.05, **p < 0.01, ***p < 0.001, ****p < 0.0001)

To determine the genomic localization of DNA modification differences between cell types, pairwise differentially modified regions (DMRs) were identified for each DNA modification type. Differential mCG regions (DMCGRs) consisted of both hyper- and hypo-methylation (Fig. 7A) and were primarily comparison-specific, with the greatest overlap being between glia (microglia and astrocytes) and neurons (Fig. 7B,C). DMCGRs were distributed across the genome for each comparison and ranged from –100 to 100% difference (Fig. 7D–F).

To analyze the localization of DMCGRs within genic contexts, over- and under-representation analysis was performed (as compared to random distribution across the background). DMCGRs for all three comparisons were over-represented in gene body regions, distal promoters, and intergenic contexts, while being under-represented in proximal promoters (Fig. 7G–I). Despite being over-represented within most cell types, astrocytic hypermethylation within distal intergenic regions compared to microglia was under-represented, demonstrating a different epigenomic patterning between individual glial cell types than between glia and neurons.

HOMER analysis was performed on DMCGRs to identify enriched motifs for each comparison (Additional file 6) [61], which revealed transcription factor binding motifs associated with cell type-specific functions. For instance, highly methylated astrocytic regions were enriched for HIF1b binding sites, while highly methylated neuron regions are enriched for Zic binding sites (Fig. 7J). These transcription factors, along with their targeted genes *Npas4* and *Apoe*, have known roles in excitatory-inhibitory balance in the central nervous system [62, 63], and lipid transport in cerebellar astrocytes [64, 65], respectively. As previously mentioned, mCG hypermethylation is generally associated with transcriptional repression. Thus, hypermethylation of these essential transcription factors have downstream implications for cell type-specific functions of CNS cells.

In the comparison between microglia and neurons (Fig. 7K), hypermethylated microglial regions were found to be enriched in Lhx2 binding sites, which inhibit *Gfap* expression and promote neurogenesis in the hippocampus [66]. Hypermethylated neuronal regions were enriched in Fli1 binding sites, which are implicated in the shift from homeostatic to ramified microglia through *Spi1* and *Runx1* [67–69]. In the comparison between astrocytic and microglial DMCGRs (Fig. 7L), hypermethylated astrocytic regions were enriched in NF1-halfsite binding sites, which has downstream regulators such as *Sp1*, *Mef2c*, and *Sall1*, all essential modulators of homeostatic microglia [70]. Microglial hypermethylation was enriched in PU.1 binding sites, and although is most well-known for its function as a master regulator of microglia, regulates the astrocytic maturation marker *Runx2* during development as well [69]. Together, motifs in DMCGRs followed the expected inverse relationship with binding sites of known cell identity-related transcription factors.

**Fig. 8:**
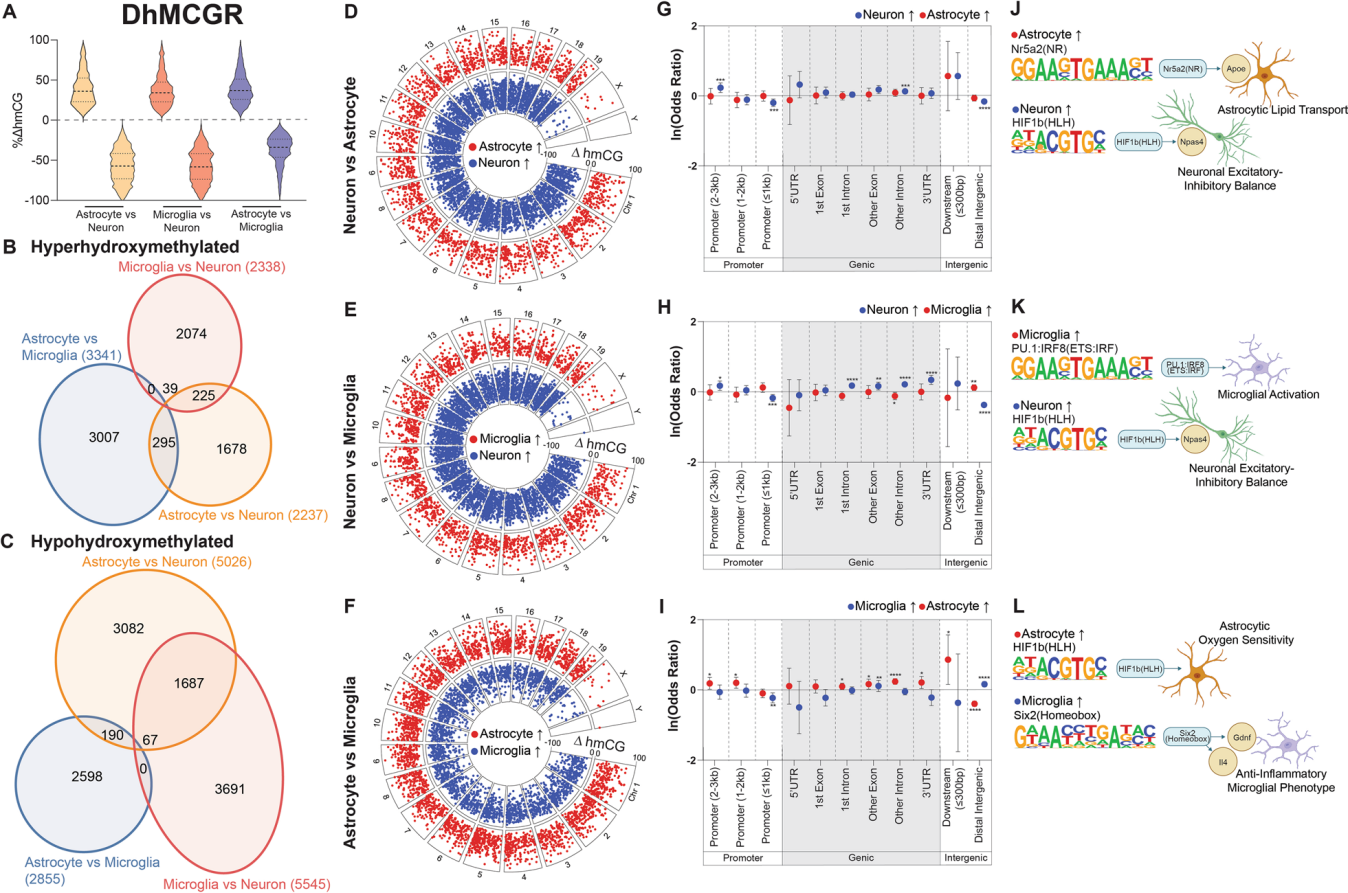
Differentially hydroxymethylated CG regions. Differentially hydroxymethylated CG regions (DhMCGRs) were determined between cell types. Distribution of hmCG differences **A** was plotted, along with overlap of hyper- **B** and hypo- **C** DhMCGRs between the three comparisons. Genomic distribution and magnitude of DhMCGRs for astrocytes vs neurons **D**, microglia vs neurons **E**, and astrocytes vs microglia **F**. Relative over- and under-representation in genic features for astrocytes vs neurons **G**, microglia vs neurons **H**, and astrocytes vs microglia **I**. Top enriched transcription factor binding motifs for astrocytes vs neurons **J**, microglia vs neurons **K**, and astrocytes vs microglia **L**. Genic regions containing no DhMCGRs were notated as “*n.d.*” (Woolf logit method for 95% confidence intervals, Fisher’s exact test for two-sided p-values; *p < 0.05, **p < 0.01, ***p < 0.001, ****p < 0.0001)

Differential hydroxymethylated CG regions (DhMCGRs) between cell types consisted of both hyper- and hypo-hydroxymethylation (Fig. 8A), and were mainly comparison-specific. The greatest overlap was in hypo-hydroxymethylated regions between glial cells and neurons (Fig. 8B, C), and DhMCGRs were distributed throughout the genome for all three comparisons (Fig. 8D–F). Analysis of the genomic distribution of DhMCGRs revealed over-representation in genic regions and distal promoter regions, with under-representation in proximal promoter regions across all comparisons (Fig. 8G–I). Specifically, neuronal and astrocytic hyper hydroxymethylation was under-represented in distal intergenic regions, whereas over-representation was observed in microglia (Fig. 8G–I).

HOMER analysis identified enriched motifs within DhMCGRs, with corresponding cell type-specific functions (Additional file 7). Specifically, when comparing astrocytes and neurons (Fig. 8J), hyper-hydroxymethylated astrocytic regions were enriched for Nr5a2 binding sites, while neuronal hyper-hydroxymethylated regions were enriched for HIF1b binding sites. Increased hydroxymethylation has been hypothesized to “functionally demethylate” specific genomic regions and positively correlate with gene expression [4]. With this in mind, hyper-hydroxymethylation of Nr5a2 (upstream of *Apoe*), in astrocytes likely induces lipid transport pathways, an essential astrocytic function [71]. Similarly, neuronal hyper-hydroxymethylation of HIF1b likely induces excitatory-inhibitory balance functions within neurons [62, 63]. Between microglia and neurons (Fig. 8K), microglial hyper-hydroxymethylation of PU.1:IRF8 binding sites is likely involved in microglial activation programming [72]. When comparing astrocytes and microglia (Fig. 8L), astrocytic hyper-hydroxymethylation was enriched in HIF1b binding sites, which may be needed for the central regulation of oxygen sensing, an important function of astrocytes [73]. Microglial hyper-hydroxymethylation was enriched in Six2 binding sites, which regulates an anti-inflammatory phenotype in microglia through *Gdnf* and *Il4* [74]. Taken together, the consistency of hyper-hydroxymethylated transcription factor binding motifs with specific cell type implications further bolster the hypothesis that hydroxymethylation serves to “functionally demethylate” specific regions of the genome that are needed for cellular identity.

**Fig. 9:**
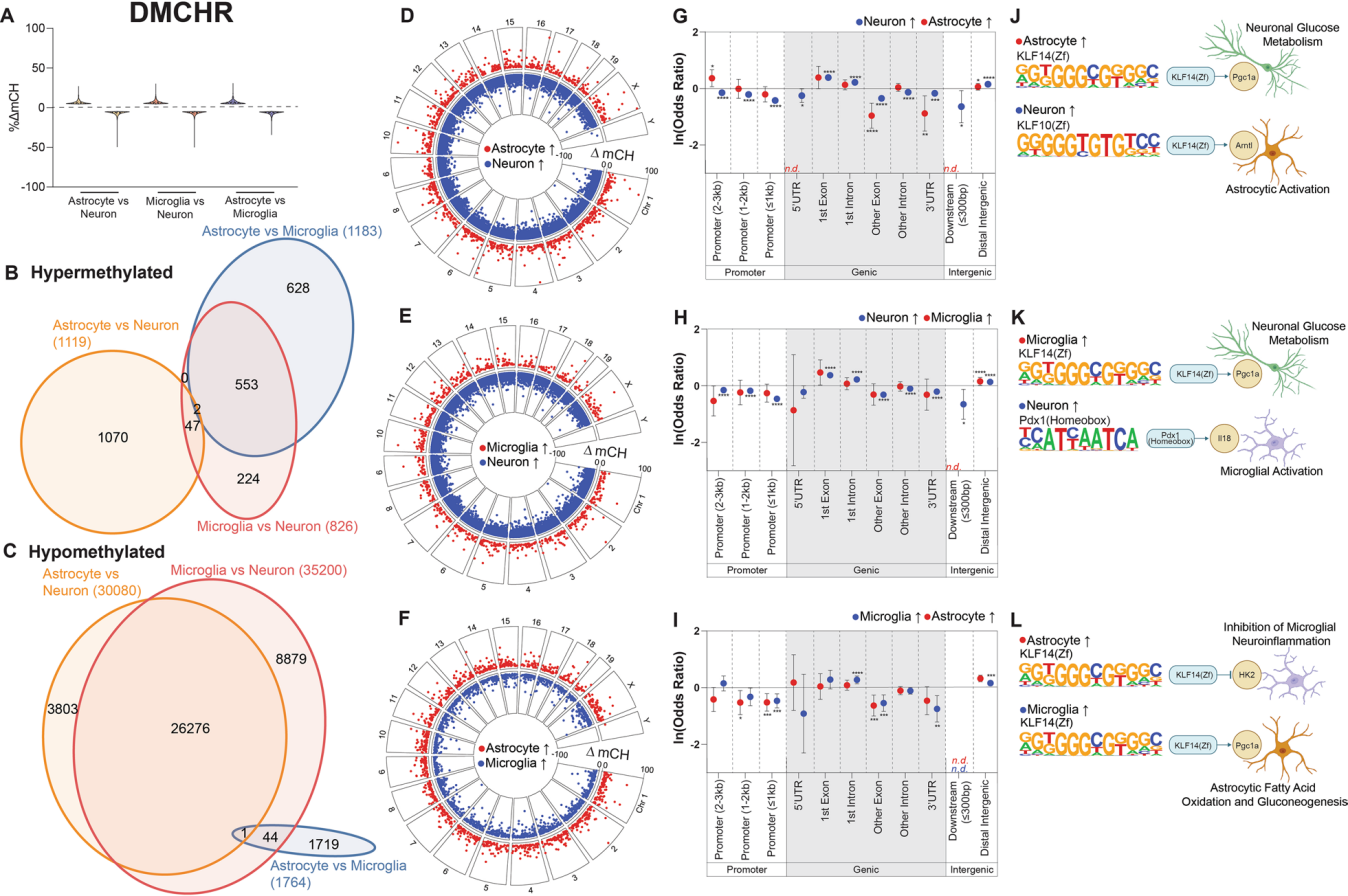
Differentially methylated CH regions**.** Differentially methylated CH regions (DMCHRs) were determined between cell types. Distribution of mCH differences **A** was plotted, along with overlap of hyper- **B** and hypo- **C** DMCHRs between the three comparisons. Genomic distribution and magnitude of DMCHRs for astrocytes vs neurons **D**, microglia vs neurons **E**, and astrocytes vs microglia **F**. Relative over- and under-representation in genic features for astrocytes vs neurons **G**, microglia vs neurons **H**, and astrocytes vs microglia **I**. Top enriched transcription factor binding motifs for astrocytes vs neurons **J**, microglia vs neurons **K**, and astrocytes vs microglia **L**. Genic regions containing no DMCHRs were notated as “*n.d.*” (Woolf logit method for 95% confidence intervals, Fisher’s exact test for two-sided p-values; *p < 0.05, **p < 0.01, ***p < 0.001, ****p < 0.0001)

Differentially methylated CH regions (DMCHRs) consisted of both hyper- and hypo-methylation (Fig. 9A). Similar to other differential modifications DMCHRs were predominantly comparison-specific, with the largest overlap observed between the microglia vs neuron and microglia vs astrocyte comparisons (Fig. 9B). Additionally, the majority of DMCHRs were shared between glial comparisons with neurons (Fig. 9C). As with the other modification types analyzed, DMCHRs were distributed across the genome, however, their magnitude tended to be smaller than CG modification differences (Fig. 9A,D–F).

Within all cell type comparisons, DMCHRs were over-represented in first exons and first introns, as well as distal intergenic regions (Fig. 9G–I). Conversely, promoter regions and later gene body regions were under-represented for DMCHRs (Fig. 9G–I). HOMER analysis identified enriched transcription factor binding sites within DMCHRs, which were associated with cell-type specific functions (Additional file 8). mCH has been shown to have an inverse relationship with expression [2], and this is demonstrated by the cell type-specific downstream functions of enriched binding sites in DMCHRs. In particular, between astrocytes and neurons, high astrocytic mCH regions were enriched for Klf14 binding sites, and high neuronal mCH regions were enriched in Klf10 binding sites (Fig. 9J). Klf14 is upstream of *Pgc1a*, an important regulator of glucose metabolism in neurons [75]. Thus, hypo-CH methylation of Klf14 in neurons is consistent with its activation in this cell type and hyper-methylation in astrocytes is consistent with repression. Klf10 is upstream of *Arntl*, a regulator of astrocytic activation [76], and astrocytic hypo-CH methylation of Klf10 binding sites is consistent with utilization of this transcription factor in astrocytes.

Similarly, when comparing microglia and neurons (Fig. 9K), high microglial mCH was enriched in Klf14 binding sites [75]. Regions of high neuronal mCH were enriched within Pdx1 binding sites, and while being most well-known for its function in the pancreas, Pdx1 has recently been recognized for its necessity in immune cells like microglia [77]. In this case, Pdx1 interacts with *Il18*, which has increased expression during microglial activation [78]. Thus, neuronal hyper-methylation of Pdx1 likely serves to repress this microglial program.

Interestingly, when comparing astrocytes and microglia, Klf14 binding sites were enriched in regions of both astrocytic and microglial hyper-CH methylation (Fig. 9L). *H2K* is a mediator of microglial activation, which can be inhibited by Klf14 [79, 80]. Klf14 also activates *Pgc1a*, which helps to facilitate fatty acid oxidation and gluconeogenesis, both important functions of astrocytes [75]. This demonstrates differential functions for the Klf14 transcription factor in multiple CNS cell types. These findings highlight the cell type-specific functions associated with differentially methylated CH regions (DMCHRs), and give further evidence that mCH contributes to gene expression regulation not only in neurons [81] but glial cells as well.

Overall, differential DNA modifications between neurons, astrocytes, and microglia are principally located within the gene body and in distal intergenic regions, not at proximal promoters as may be expected, and have enriched transcription factor binding motifs with downstream cell type-specific functions.

### Verification of differentially CG hydroxymethylated regions with targeted EM-Seq

To validate the differentially CG hydroxymethylated regions identified with WGoxBS, targeted enzymatic methyl sequencing (EM-seq) was performed using INTACT-isolated gDNA from neurons, astrocytes, and microglia (*n* = 3/group). Unlike WGoxBS which uses a chemical conversion with sodium bisulfite, EM-seq enzymatically converts DNA with TET2. Deamination following conversion results in the detection of total modifications (mC + hmC). Performing a mock conversion reaction with no TET2 followed by deamination allows for the direct detection of hmC (Additional file 10: Figure S2A–B). Six regions were selected that had both large hmCG differences between two cell types and were located within a gene or gene regulatory element that showed a significant difference in expression between cell types with a reported cell type-specific function (Additional file 1C). *Chn1*, *Dlgap1*, *Ankrd33b*, *Dab2ip*, and *Kalrn* are highly expressed in neurons and have functions related to signal transduction [82], postsynaptic scaffolding [83], binding of neuronal-specific calcium-binding proteins [84], neuronal migration [85], and synaptic function [86], respectively. *Chst2* is highly expressed in astrocytes and has a predicted function in astrocyte reactivity [87].

**Fig. 10:**
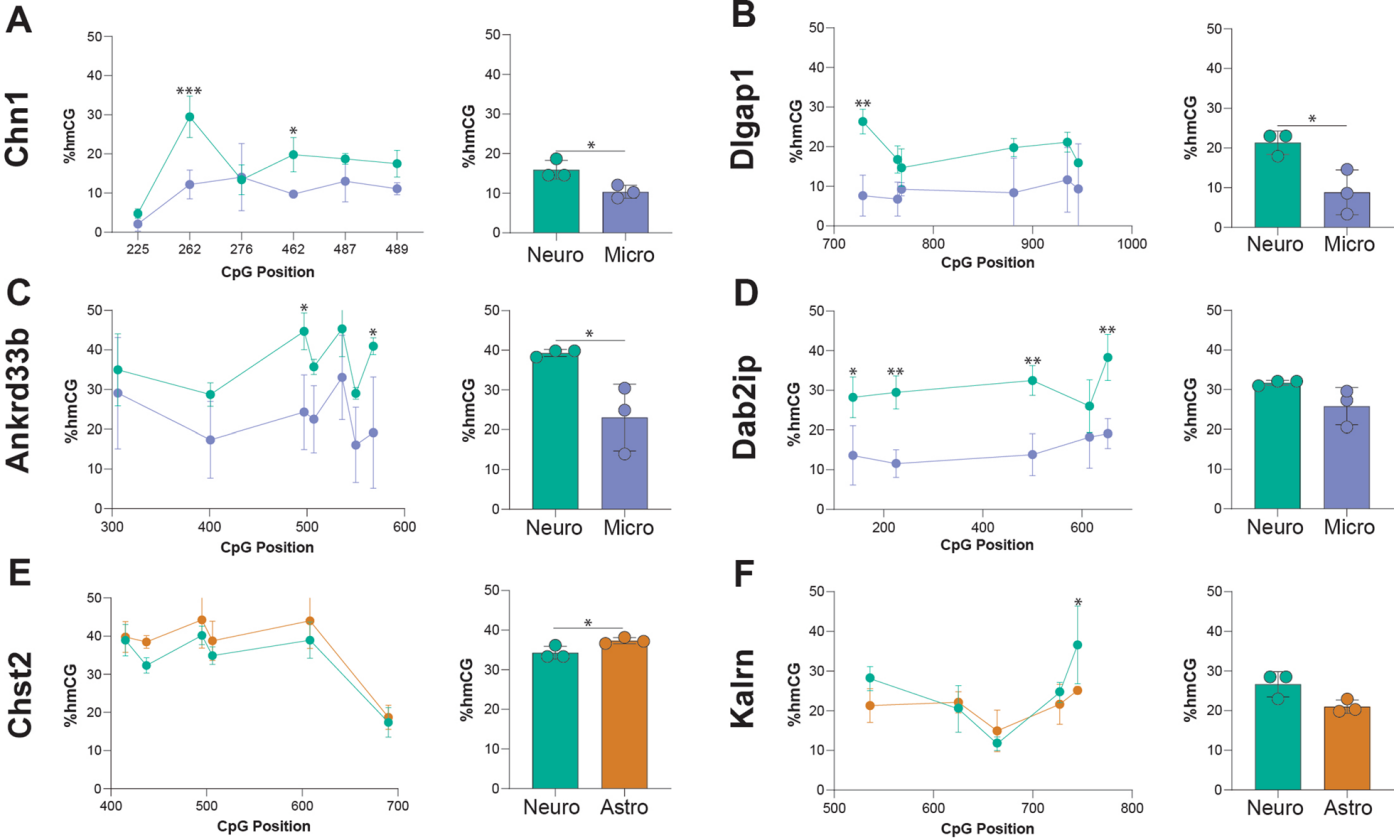
Verification of differentially hydroxymethylated regions. Targeted enzymatic methyl-seq (EM-seq) was performed from INTACT-isolated gDNA from neurons, astrocytes, and microglia (*n *= 3/group) in six regions found to be differentially hydroxymethylated with oxBS. Line plots and total hmCG were plotted for regions corresponding to *Chn1* **A**, *Dlgap1* **B**, *Ankrd33b* **C**, *Dab2ip* **D**, *Chst2* **E**, and *Kalrn* **F **(two-way ANOVA with Sidak’s multiple testing correction and single pooled variance for individual CpG differences between cell types, two-tailed unpaired t-test for average region differences between cell types; *p < 0.05, **p < 0.01, ***p < 0.001)

Of the six regions assessed, four showed significant differences in hydroxymethylation between cell types across the entire 1000 bp region: *Chn1* (Fig. 10A), *Dlgap1* (Fig. 10B), *Ankrd33b* (Fig. 10C) between neurons and microglia, and *Chst2* (Fig. 10E) between astrocytes and neurons. While *Dab2ip* (Fig. 10D) and *Kalrn* (Fig. 10F) did not show statistically significant differences in hmCG across the entire region, there are statistically significant differences at individual CpG positions. Nonetheless, this targeted region analysis in independent samples with a different conversion chemistry and very high coverage (> 4000X) recapitulated the WGoxBS findings. CG methylation values in these regions were also significantly different between cell types both across the entire 1000 bp region and at specific CpG sites, with the exception of *Dlgap1* (Additional file 10: Figure S6). Since mCG was higher in the opposite cell type as hmCG (Additional file 10: Figure S6), the correlation between DMCGRs and DhMCGRs was assessed. DMCGRs and DhMCGRs have a significant inverse correlation with one another (Additional file 10: Figure S7), suggesting that there is an exchange of these two modifications in differentially modified regions of the genome.

### Relationship of DNA modifications to gene expression is conserved across three CNS cell types

**Fig. 11:**
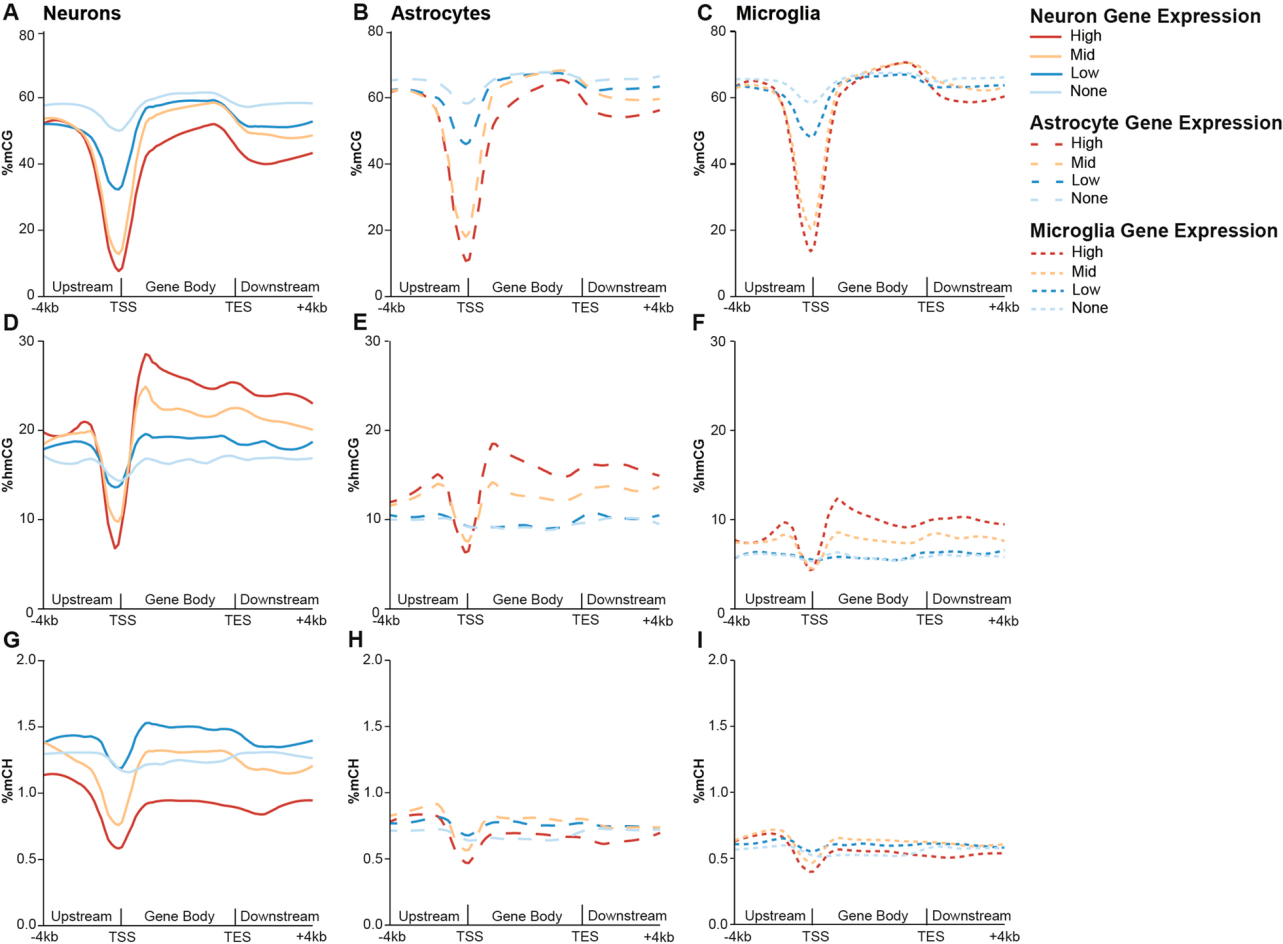
Relationship of DNA modifications to gene expression is conserved across CNS cell types. High, mid, low, and unexpressed genes were identified for each cell type from RNA-seq data. Percent mCG **A–C**, hmCG **D–F** and mCH **G–I** averaged over 200 nucleotide bins from 4 kb upstream to 4 kb downstream of genes based on their expression level in each cell type

To examine the relationship between DNA modifications and gene expression in the mouse brain at a cell type-specific level, we performed analyses of positive fraction Camk2a-NuTRAP, Aldh1l1-NuTRAP, and Cx3cr1-NuTRAP paired TRAP-RNA-seq (GSE140895, present study— GSE228043) and INTACT-WGoxBS-seq (GSE140271, present study—GSE228044) data. For each cell type, genes were separated into groups of unexpressed and expressed genes, with expressed genes being further divided into high, mid, and low expressed tertiles. Average mCG, hmCG, and mCH levels were plotted 4 kb upstream, within the gene body, and 4 kb downstream of those genes. As gene expression is different between cell types, the composition of the individual lists is cell type specific (Additional file 10: Figure S7; Additional file 9). Across cell types, an inverse relationship between expression and mCG was observed at the TSS, as expected, and this relationship was maintained upstream, throughout the gene body, as well as downstream of the gene body (Fig. 11A–C). Hydroxymethylation, while having an inverse relationship with expression at the TSS, demonstrated a positive relationship with expression within the gene body of all cell types analyzed (Fig. 11D–F). Notably, lowly expressed neuronal genes exhibited the highest level of mCH (Fig. 11G), which has been previously described as a mechanism for fine-tuning post-developmental gene expression through MeCP2 binding [6, 48, 88]. In contrast, mCH did not vary appreciably with gene expression in astrocytes or microglia, likely due to their low genomic mCH levels (0.5–1%) (Fig. 11H, I).

## Discussion

Epigenomic regulation plays a crucial role in determining cell type identity and phenotypic state [5], highlighting a need for tools to assess the neuro-epigenome at base- and cell type-specific resolution. Tissue-level analysis of the brain have provided important insights into epigenomic mechanisms regulating the genome [89–91], but the presence of mixed cell populations in whole tissue samples obscures the cell type-specificity of these mechanisms. Isolating specific cell types (i.e., via cell sorting) from the brain poses its own set of challenges, as a lack of adequate cell surface markers (particularly for neurons) and molecular changes that occur during the creation of a single cell suspension confound these types of cell type-specific analyses [92, 93]. Transgenic labeling approaches (such as RiboTag, TRAP, INTACT, and NuTRAP) provide the desired cell type specificity and avoid these activational confounds of sorting [92–94]. Adding to the growing arsenal of tools for neuro-epigenomic studies [41], here we validated a neuronal-specific Camk2a-NuTRAP model, which allows for isolation of paired DNA and RNA from hippocampal excitatory neurons for a robust cell type-specific granularity to the neuro-epigenomic regulation of gene expression.

Examining DNA modifications in specific cell types and subtypes is critical to understanding their roles in genome regulation and gene expression. However, cell type-specific DNA modification studies outside of neurons are limited, and what has been done is largely NeuN^+^ vs NeuN^−^ sorting [2, 28], which does not differentiate between glial (i.e., astrocytic, microglial, or oligodendrocytic) populations. Moreover, NeuN is a pan-neuronal marker [95], and considering that neuronal subtypes have different levels of mCG and hmCG [3], further granularity is needed here as well. This is not completely resolved even with the model presented here as Camk2a^+^ labeled neurons, while in a focused brain region (e.g., hippocampus), also represent a somewhat mixed population of pyramidal and granule neurons. However, the ability to temporally label specific neuronal populations enables more specific analysis of their epigenomic patterns. Single cell bisulfite sequencing has been conducted in the brain [49, 50], and while providing cell type specificity, is limited in its genomic coverage and does not currently have the sensitivity required to detect both mC and hmC.

The findings in this study emphasize the importance of distinguishing between methylation and hydroxymethylation when characterizing epigenome patterns and their relationship to gene expression. Bisulfite sequencing does not distinguish between mC and hmC [96], and data obtained through this method are often referred to as “methylation” while actually being total modifications [97]. To circumvent these issues, antibody-based methods have been employed to assess mC and hmC, and though providing insight into overall modification levels [98–101], they do not provide base-specificity and preferentially pull down CG dense regions of the genome making it difficult to assess the relationship between modifications and gene expression. Thus utilization of base-specific quantitative methods for differentiating mC versus hmC, such as oxidative bisulfite sequencing [102], enzymatic methyl-seq (EM-Seq) [103], or native reading of modifications through nanopore sequencing [104] is critical in neuroscience studies of DNA modifications. Each of the methods employed in this study (WGoxBS, EM-Seq and nanopore sequencing) has advantages but returned highly similar modification levels, adding to the confidence in the technical validity of these approaches.

In this study, we used validated NuTRAP models to examine DNA modifications in neurons, astrocytes and microglia. While total modification levels are similar across cell types, when split into methylation and hydroxymethylation mCG levels were highest in microglia, followed by astrocytes and then neurons. Conversely, hmCG and mCH levels were highest in neurons, followed by astrocytes and then microglia. This is in agreement with data identifying neurons as a primary source of hmCG and mCH in the brain [2–4, 105] but these findings also demonstrate the existence of hmCG and mCH in microglia and astrocytes that relates to gene expression levels. The presence of hmCH is still highly debated [2–4]. This modification is likely present in low amounts, if at all, and assessment with a direct readout of hmC such as EM-seq [106] would elucidate its presence and relationship to gene expression.

The expression of DNA modification regulators (DNMTs, TETs, TDG) did not have an obvious relationship to DNA modifications, suggesting the involvement of cell type-specific cofactors in driving these enzymes to particular genomic locations. Exploration of DNA modification regulation mechanisms in specific CNS cell types is an important future direction for the field.

Relative modification levels across cell types in repetitive elements followed the pattern observed at the whole genome level. Repeat elements exhibited higher mCG levels and lower hmCG levels compared to non-repeat elements, indicating a strong repressive signal for repetitive elements. Transposable elements are thought to be more active during neuronal development [107–109], and the lower levels of mCG and higher levels of hmCG in mature neurons compared to astrocytes and microglia might reflect their developmental history or leave them poised for potential reactivation, although further investigation is required to elucidate this in greater detail.

The analysis of differential modification levels between cell types in this study revealed consistent trends with total modification levels, showing specific regions of hyper- or hypo-modifications for each comparison. Across comparisons, differential modifications were enriched in genic and distal intergenic regions, while being depleted within proximal promoters. This adds to a growing body of work recognizing distal regulatory regions of the genome, and not promoters, as the key regulators of cell identity [2, 3, 50, 110, 111].

Interestingly, hypomodifications were observed at proximal promoter regions, and these modifications showed an inverse correlation with expression across cell types. hmCG then demonstrated a positive association with expression in the gene body. While this relationship has been described in neurons [3, 4, 112], the relationship of hmCG to expression has not been characterized in astrocytes and microglia and provides strong evidence that hmCG is a genome regulator in glial cell types of the CNS as well as neurons. While no discernable associations between astrocytic and microglial mCH were clear, greater sequencing depth could help resolve this.

Integration of the data presented here with chromatin accessibility, histone modifications and additional genomic features by machine learning models, will contribute to a more precise understanding of the complex epigenomic regulation of gene expression that is moving beyond simplistic associations to one that is modification and context specific. While this study focused on the hippocampus, future work using the NuTRAP models and sequencing approaches that differentiate mC from hmC can be used to assess any additional CNS regions of interest. Additionally, these models and approaches can be used to examine the dynamic nature of DNA modification patterns during development, health, and neurological disorders [113, 114] in specific CNS cell types.

## Conclusions

Here, we validate a new model for studying the neuronal epigenome that circumvents the need for cell sorting, while providing greater whole genome coverage than currently available single cell techniques. While the absolute mCG, hmCG, and mCH levels across the three CNS cell types analyzed differed, the relationship of each of these modifications to gene expression is consistent across cell types. These findings demonstrate that the relationship between DNA modifications and gene expression is dependent on the genomic context and the relative modification level for that cell rather than an absolute modification level. Integration of data such as these with chromatin landscapes should reveal a more complete understanding of gene expression regulation through epigenetic mechanisms. Furthermore, gene body and intergenic region modifications, likely at enhancers, were stronger indicators of cellular identity than promoter modifications indicating that a focus on DNA modifications in proximal promoters is too simplistic.

## Methods

### Animals

All animal procedures were approved by the Institutional Care and Use Committee at the Oklahoma Medical Research Foundation (OMRF). Mice were purchased from the Jackson Laboratory (Bar Harbor, ME), bred, and housed in the animal facility at OMRF, under SPF conditions in a HEPA barrier environment. Camk2a-cre/ERT2^+/wt^ males (stock #012362) were mated with NuTRAP^flox/wt^ females (stock #029899) to generate the desired Camk2a-Cre/ERT2^+/wt^; NuTRAP^flox/wt^ (Camk2a-cre/ERT2^+^; NuTRAP^+^) progeny. DNA was extracted from ear punch samples for genotyping. Male and female mice were ~ 4 months of age at the time of experiments. Euthanasia prior to tissue harvesting was carried out by cervical dislocation and decapitation. The primers used for genotyping (Integrated DNA Technologies, Coralville, IA) are included in Additional file 1A.

### Tamoxifen (Tam) treatment

At ~ 3 months of age, mice received a daily intraperitoneal (i.p.) injection of tamoxifen (Tam) solubilized in 100% sunflower seed oil by sonication (100 mg/kg body weight, 20 mg/mL stock solution, #T5648; Millipore Sigma, St. Louis, MO) for five consecutive days. Experiments were performed 1 month after Tamoxifen administration unless otherwise specified.

### Immunohistochemistry and imaging

Brains from either Tam-induced (Tam +) or vehicle (Tam-) treated Camk2a-cre/ERT2^−^; NuTRAP^+^ or Camk2a-cre/ERT2^+^; NuTRAP^+^ mice were harvested and hemisected. Samples were fixed for 4 h in 4% paraformaldehyde (PFA), embedded in 2% agarose, and vibratome-sectioned (Vibratome 3000 Sectioning System, The Vibratome Company, St. Louis, MO). Two-hundred μm-thick sagittal sections were permeated for 2 h in PBS containing 3% BSA and 0.2% Triton, and processed for fluorescence immunostaining. The primary antibodies used included chicken anti-mCherry (#ab205402, 1:500, Abcam), rabbit anti-NeuN (#ab177487, 1:200, Abcam), rat anti-CD11b (#C227, 1:200, Leinco Technologies, St. Louis, MO), chicken anti-GFAP (#ab4674, 1:1000, Abcam), and hamster anti-CD31 (#2H8, 1:100, Developmental Studies Hybridoma Bank). For confocal imaging of *nuclei suspensions*, unfixed, freshly isolated nuclei were mixed with DAPI solution. Sequential imaging of nuclei was performed on a Zeiss Axiobserver Z1 Fluorescence Motorized Microscope (Carl Zeiss Microscopy, LLC, White Plains, NY) at the OMRF Imaging Core Facility. Microscope and software (Zen Black 3.1) settings were identical/similar for all samples, capture at 40X magnification. For *brain vibratome sections*, imaging was performed on an Olympus FluoView confocal laser-scanning microscope (FV1200; Olympus; Center Valley, PA) at the Dean McGee Eye Institute imaging core facility at OUHSC. Microscope and FLUOVIEW FV1000 Ver. 1.2.6.0 software (Olympus) settings were identical for samples within experiments at same magnification. The experimental format files were.oif or.oib. The final Z-stack generated was achieved at 1.22 µm step size with a total of 20 optical slices at 20X magnification (1X zoom) (Fig. 1), 1.26 µm step size with a total of 22 optical slices at 20 X magnifications (1.5X zoom) (Additional file 10: Figure S1A,B), and 0.62 μm step size with a total of 32–50 optical slices at 40X magnification (1X zoom) (Additional file 10**:** Figure S1C–E). For all confocal images, raw files were exported as TIFF files for downstream processing and figure assembly in Adobe Photoshop V: 24.5.0 (Adobe Photoshop).

### Translating ribosome affinity purification (TRAP) and RNA extraction

The purification of cell-specific RNA from Tam-induced Camk2a-NuTRAP mice (*n* = 4) was achieved by following an established protocol [41]. One hippocampal hemisphere was minced into small pieces and homogenized in 100μL ice-cold homogenization buffer (50 mM Tris, pH 7.4; 12 mM MgCl_2_; 100 mM KCl; 1% NP-40; 1 mg/mL sodium heparin; 1 mM DTT) supplemented with 100 μg/mL cycloheximide (#C4859-1ML, Millipore Sigma), 200 units/mL RNaseOUT^™^ Recombinant Ribonuclease Inhibitor (#10,777,019; ThermoFisher), and 1X cOmplete, EDTA-free Protease Inhibitor Cocktail (#11,836,170,001; Millipore Sigma) with a pellet pestle cordless motor (Kimble) with one 10 s pulse. 300 μL ice-cold homogenization buffer was added and homogenized again with one 10 s pulse and volume brought to 1.5 mL with homogenization buffer. The homogenate was transferred to a 2 mL round-bottom tube and centrifuged at 12,000 ×g for 10 min at 4 °C. After centrifugation, 100 μL of the supernatant was saved as the input. The remaining supernatant was transferred to a 2 mL round-bottom tube and incubated with 5 μg/μL of anti-GFP antibody (ab290; Abcam) at 4 °C with end-over-end rotation for one hour. Dynabeads Protein G for Immunoprecipitation (#10003D; ThermoFisher) were washed three times in 1 mL ice-cold low-salt wash buffer (50 mM Tris, pH 7.5; 12 mM MgCl_2_; 100 mM KCl; 1% NP-40; 100 μg/mL cycloheximide; 1 mM DTT). After the last wash, 30 μL of washed Protein-G Dynabeads were added to the homogenate/antibody mixture and incubated at 4 °C with end-over-end rotation overnight. Magnetic beads were collected using a DynaMag-2 magnet and the unbound-ribosomes and associated RNA saved as the “negative” fraction (depleted). Beads were then washed three times with 1 mL of high-salt wash buffer (50 mM Tris, pH 7.5; 12 mM MgCl_2_; 300 mM KCl; 1% NP-40; 100 μg/mL cycloheximide; 2 mM DTT). Following the last wash, 350 μL of Buffer RLT (Qiagen) supplemented with 3.5 μL 2-β mercaptoethanol was added directly to the beads and incubated with mixing on a ThermoMixer (Eppendorf) for 10 min at room temperature. The beads were magnetically separated and the supernatant containing the target bead-bound ribosomes and associated RNA was transferred to a new tube. 350 μL of 100% ethanol was added to the tube (positive fractions: enriched in transcriptome associated to EGFP-tagged ribosomes) and then loaded onto a RNeasy MinElute column. RNA was isolated using RNeasy Mini Kit (#74,104, Qiagen), according to manufacturer’s instructions. RNA was quantified with a Nanodrop 2000c spectrophotometer (ThermoFisher Scientific) and its quality assessed by HS RNA screentape with a 2200 Tapestation analyzer (Agilent Technologies).

### Quantitative PCR (qPCR)

Targeted gene expression analysis was performed with qPCR. cDNA was synthesized with the ABI High-Capacity Reverse Transcription Kit (Applied Biosystems Inc., Foster City, CA) from 25 ng of purified RNA. qPCR was performed with gene-specific primer probe fluorogenic exonuclease assays (TaqMan, Life Technologies, Waltham, MA, Additional file 1**B**) and the QuantStudio^™^ 12 K Flex Real-Time PCR System (Applied Biosystems). Relative gene expression (RQ) was calculated with Expression Suite v 1.0.3 software using the 2^−ΔΔ^Ct analysis method with *Gapdh* as an endogenous control.

### Library construction and RNA sequencing (RNA-seq)

The NEBNext Ultra II Directional Library Prep Kit for Illumina (#NEBE7760L; New England Biolabs Inc., Ipswich, MA) was used on 25 ng of total RNA for the preparation of strand-specific sequencing libraries from input, negative, and positive fractions of each TRAP-isolated RNA sample according to manufacturer’s instructions. Briefly, polyA containing mRNA was purified using oligo-dT attached magnetic beads. mRNA was chemically fragmented and cDNA synthesized. For strand specificity, the incorporation of dUTP instead of dTTP in the second strand cDNA synthesis does not allow amplification past this dUTP with the polymerase. Following cDNA synthesis each product underwent end repair process, the additional of a single ‘A’ base, and finally ligation of adapters. The cDNA products were further purified and enriched using PCR to make the final library for sequencing. Library sizing was performed with HS D1000 screentape (#5067-5582; Agilent Technologies) and libraries were quantified using Qubit dsDNA HS Assay Kit (ThermoFisher Scientific). The libraries for each sample were pooled at 4 nM concentration and sequenced using an Illumina NextSeq 550 (PE 75 bp) at the Oklahoma Medical Research Foundation Clinical Genomics Core Facility.

### RNA-seq data analysis

Following sequencing, reads were trimmed, aligned, differential expression statistics and correlation analyses were performed in Strand NGS software package (Agilent) [115]. Reads were aligned against the Mm10 build of the mouse genome (2014.11.26). Alignment and filtering criteria included: adapter trimming, fixed 2 bp trim from 5’ and 6 bp from 3’ ends, a maximum number of one novel splice allowed per read, a minimum of 90% identity with the reference sequence, a maximum of 5% gap, trimming of 3′ end with Q < 30. Alignment was performed directionally with Read 1 aligned in reverse and Read 2 in forward orientation. Reads were filtered based on the mapping status and only those reads that aligned normally (in the appropriate direction) were retained. Normalization was performed with the DESeq algorithm [116]. Transcripts with an average read count value > 20 in at least 100% of the samples in at least one group were considered expressed at a level sufficient for quantitation and those transcripts below this level were considered not detected/not expressed and excluded, as these low levels of reads are close to background and are highly variable. For statistical analysis of differential expression, a one-way ANOVA was performed using the factor of TRAP fraction, and a Benjamini–Hochberg Multiple Testing Correction followed by Student–Newman–Keuls post hoc test. For those transcripts meeting this statistical criterion, a fold change >|1.25| cutoff was used to eliminate those genes which were statistically significant but unlikely to be biologically significant and orthogonally confirmable due to their very small magnitude of change. Visualizations of hierarchical clustering and principal component analysis were performed in Strand Next Generation Analysis Software (NGS) (Version 4.0, Bangalore, India). The entirety of the sequencing data is available for download in FASTQ format from NCBI Sequence Read Archive (GSE228045). Cell type specific marker gene lists were generated from the re-analysis of lists published by McKenzie et al. [117] of immunopurified [118] and high throughput single cell data from mice [119, 120]. Published lists were filtered first by mean enrichment score of ≥ 3.5 and secondly to remove any genes that appeared on lists for multiple cell types. Cell population estimates within each fraction were calculated using CIBERSORTx [45], provided with raw RNA-sequencing data for each sample and cell type marker lists described above. Briefly, single-cell RNA-seq data [118] were reformatted according to the requirements of CIBERSORTx. A signature matrix was created from those data using default settings. The cellularity of each sample from TRAP RNA-seq (input, negative, and positive fractions) was imputed using the signature matrix reference and default settings. Enriched and depleted genes were imported into the Ingenuity Pathway Analysis (IPA) software (Version 01.12, Qiagen Bioinformatics) and Gene Ontology Enrichment Analysis to assess pathway/biological function enrichment, as well as identify biological processes enriched and depleted in the positive fraction compared to input.

### Isolation of nuclei tagged in specific cell types (INTACT), and gDNA and nuclear RNA extraction

Purification of viable, cell type-specific nuclei from Tam-induced Camk2a-NuTRAP mouse hippocampus (*n* = 4) was achieved by combining two previously published protocols [32, 121] with modifications as described previously [41]. One hippocampal hemisphere from the contralateral side as TRAP isolation was rinsed in ice-cold 1X PBS, minced into small pieces, and homogenized in 1 mL ice-cold nuclei EZ lysis buffer (#NUC-101, Millipore Sigma) supplemented with 1X Halt protease inhibitor cocktail (ThermoFisher Scientific) using a glass dounce tissue grinder set (#D9063; Millipore Sigma; 20 times with pestle A and 20 times with pestle B). Undissociated tissue, largely composed of blood vessels, was removed by centrifugation at 200 ×*g* for 1.5 min at 4 °C, and the supernatant containing the nuclear material filtered through a 30 μm strainer and centrifuged at 500 × g for 5 min at 4 °C. The resulting nuclear pellet was resuspended in nuclei lysis EZ buffer, incubated on ice for 5 min, washed by centrifugation, and resuspended in 200 μL nuclei EZ storage buffer by gentle trituration with a micropipette. From the total resuspended pellet volume, 10% was reserved as input nuclei fraction and the rest was diluted with 1.6 mL nuclei purification buffer (NPB: 20 mM HEPES, 40 mM NaCl, 90 mM EDTA, 0.5 mM EGTA, 1X Halt protease inhibitor cocktail), and subjected to the INTACT protocol. Briefly, 30 μL of resuspended M-280 Streptavidin Dynabeads (#11,205, ThermoFisher Scientific) were added into a fresh 2 mL microcentrifuge tube and washed with 1 mL of NPB using a DynaMag-2 magnet (#12,321; ThermoFisher Scientific) for a total of three washes (1 min incubation/each). The washed beads were reconstituted to their initial volume (30 μL) with NPB and gently mixed with the nuclear suspension. The mixture of nuclei and magnetic beads was incubated at 4 °C for 40 min under gentle rotation settings to allow the affinity binding of streptavidin beads to the cell-specific, biotinylated nuclei. After incubation, the streptavidin-bound nuclei were magnetically separated with the DynaMag-2 magnet for a period of 3 min and the unbound nuclei collected in a fresh 2 mL microcentrifuge tube, centrifuged at 4 °C (1000 ×*g*, 3 min), resuspended in 100 μL of NPB and reserved as the negative nuclei fraction. The nuclei bound to the beads were washed in the magnet for three washes (1 min/each), resuspended in 30 μL of NPB, and reserved as the positive nuclei fraction. From each nuclear fraction [input, negative (depleted of biotinylated nuclei), and positive (enriched in biotinylated nuclei)], a 3 μL aliquot was mixed with equal volume of DAPI counterstain and used for confocal microscopy visualization and calculation of purity percentage (3–5 fields of view per sample). The AllPrep DNA/RNA Micro Kit (#80,284, Qiagen, Germantown, MD) was used to extract gDNA and nuclear RNA for each sample. gDNA and nucRNA were quantified using a Nanodrop 2000c spectrophotometer (ThermoFisher Scientific) and its quality assessed by genomic DNA D1000 (#5067–5582) and High Sensitivity RNA (#5067–5579) screentapes with a 2200 Tapestation analyzer (Agilent Technologies, Santa Clara, CA).

### Library construction and oxidative bisulfite sequencing (OxBS-seq)

For each input, negative, and positive INTACT-isolated sample, 400 ng of gDNA was brought up to 50 μL volume with 1X low-EDTA TE buffer and sheared with a Covaris E220 sonicator (Covaris, Inc., Woburn, MA) to an average 200 base pare size using the following settings: intensity of 5, duty cycle of 10%, 200 cycles per burst, 2 cycles of 60 s, at 7 °C. The size of sheared products was confirmed by capillary electrophoresis (DNA D1000 Agilent). gDNA fragments were cleaned by Agencourt bead-based purification protocol, after which gDNA was quantified (Qubit^™^ dsDNA ThermoFisher Scientific). Two aliquots of 200 ng gDNA fragments were prepared in a 12 μL volume to which 1 μL of spike-in control DNA (0.08 ng/μL) with known levels of specific mC, hmC, and fC at individual sites was added. End repair, ligation of methylated adaptors (#L2V11DR-BC 1-96 adaptor plate, NuGEN, Tecan Genomics, Inc., Redwood City, CA) and final repair were performed according to manufacturer’s instructions (Ovation Ultralow Methyl-Seq Library System, NuGEN). Of the two DNA aliquots per sample, one was oxidized and then bisulfite-converted and the other only bisulfite converted with the True Methyl oxBS module (NuGEN) with desulfonation and purification. qPCR was performed to determine the number of PCR cycles required for library amplification. Bisulfite and oxidative bisulfite-converted samples were both amplified for 17 cycles [95 °C−2 min, N (95 °C−15 s, 60 °C−1 min, 72 °C−30 s)]. Amplified libraries were purified with Agencourt beads and eluted in low-EDTA TE buffer. Tapestation HS D1000 was used to validate and quantify libraries. Amplified libraries were normalized to a concentration of 4 nM and pooled, denatured, and diluted to 12 pM for initial QC sequencing on the MiSeq, followed by deeper sequencing of the input and positive fraction on the NovaSeq 6000 (Illumina). This was done according to manufacturer’s guidelines with the exception of a custom sequencing primer (MetSeq Primer) that was spiked in with the Illumina Read 1 primer to a final concentration of 0.5 μM.

### OxBS-seq data analysis

Prior to alignment, paired-end reads were adaptor-trimmed and filtered using Trimmomatic 0.35. End-trimming removed leading and trailing bases with a Q-score < 25, cropped 5 bases from the start of the read, dropped reads less than 30 bases long, and dropped reads with average Q-score < 25. Alignment of trimmed bisulfite converted sequences was carried out using Bismark 0.16.3 with Bowtie 2 against the soft-masked mouse reference genome (GRCm38/mm10). BAMs were de-duplicated with Bismark. Methylation call percentages for each CpG and non-CpG (CH) site within the genome were calculated by dividing the methylated counts over the total counts for that site in the oxidative bisulfite-converted libraries (OxBS). Genome-wide CpG and CH methylation levels were calculated separately. Hydroxymethylation levels in CpG (hmCG) and CH (hmCH) contexts were calculated by subtracting call levels from the oxidative bisulfite-converted libraries from the bisulfite-converted libraries. De-duplicated BAM files were run through methylKit in R to generate context-specific (CpG/CH) coverage text files [122]. Bisulfite conversion efficiency for C, mC, and hmC was estimated using CEGX spike-in control sequences. Untrimmed fastq files were run through CEGX QC v0.2, which output a fastqc_data.txt file containing the conversion mean for C, mC, and hmC. A conversion correction was performed on whole genome levels of mCG and hmCG based on the conversion efficiency calculated from CEGX spike-in controls using the equations provided in Kozlenkov et al. [3]. These are provided in Additional file 10**: **Table S1, but were not used in subsequent analyses. Analysis of methylation levels in the proximity of the promoter region was performed on a list of selected genes as follows. The R package EnrichedHeatmap was used to intersect methylation call files with genomic coordinates of gene lists [123]. Flanking regions of 4000 nucleotides were constructed upstream of the transcription start site (TSS) and downstream of the transcription end site (TES) and then split into 20 bins of 200 nucleotides each. The gene body was split into 27 equal bins, depending on the gene length. The average of each bin for all genes in the list was then plotted versus the bin number using the R package ggplot2 to give a visualization of the overall pattern of mCG, hmCG, and mCH within and around all genes contained in the gene lists [124]. Average mCG, hmCG, and mCH levels were calculated for the upstream region (− 4 kb to TSS), gene body (TSS to TES), and downstream region (TES to + 4 kb) for each gene list and biological replicate, and subjected to 2-way ANOVA statistical analysis with Sidak’s multiple comparisons correction (GSE228044).

### Nanopore long read sequencing and native 5mC and 5hmC calling

The INTACT protocol was adapted to isolate high molecular-weight gDNA from the whole brain of Camk2a-NuTRAP, Aldh1l1-NuTRAP, and Cx3cr1-NuTRAP mice (*n* = 2, 12mo) for native 5mC and 5hmC calling from Nanopore long read sequencing. Briefly, following INTACT-isolation, high molecular-weight gDNA was isolated from the positive fraction using the MagAttract HMW DNA Kit (#67,563, Qiagen) following the manufacturer’s protocol for isolation from fresh tissue. For each biological replicate, 1 µg of gDNA was used to prepare nanopore sequencing libraries using the Ligation Sequencing Kit V14 (SQK-LSK114, Oxford Nanopore Technologies) following the manufacturer’s protocol. Libraries were sequenced on PromethION R10.4.1 flow cells with a 5 kHz sampling rate. Canonical bases, mC, and hmC were called in Guppy (guppyv6.5.7) using the dna_r10.4.1_e8.2_400bps_5khz_modbases_5hmC_5mC_cg_sup_prom.cfg configuration, and read splitting was enabled. Reads were aligned to the mm10 genome assembly with minimap2, and coordinate sorting and indexing of modified-base bam files performed with samtools. Modkit (https://github.com/nanoporetech/modkit) was used to quantify genome-wide modifications for each sample using a 0.95 quality filter threshold for all CpG sites mapped to the reference genome. A one-way ANOVA was performed to assess whole genome modification differences between cell types. Chromosome-scale plots of mC and hmC within each replicate were generated with methylartist [125]. Methylartist was also used to generate violin plots of repeat element (LINE, SINE, and LTR) mCG and hmCG (PRJNA1026932).

### Transposable element modification analysis

Using the Bismark alignment of WGoxBS-Seq data to a soft-masked mm10 genome described previously, transposable element (TE) mCG, hmCG, and mCH was also examined. RepeatMasker BED files were obtained from the UCSC Genome Browser Table Browser (http://genome.ucsc.edu) [126], and modification levels were assessed in repeat and non-repeat regions of the genome, as well as within specific repeat elements (LINE, SINE, LTR, and Simple repeats). The context-specific CpG/CH MethylKit text files (described previously) were first intersected with the whole RepeatMasker BED file using ‘bedtools intersect’ with both the -wo and -v arguments to assess modification levels in repeats and non-repeats, respectively. A two-way ANOVA with Tukey’s multiple comparisons correction was performed. Next, BED files for individual classes of repeat elements (LINE, SINE, LTR, and Simple repeats) were intersected with context-specific CpG/CH methylKit text files (described previously) to assess modification levels within specific repeat elements. A one-way ANOVA with Tukey’s multiple comparisons correction was performed.

### DMCGR and DMCHR analysis

CpG and CH text files were read into methylKit [122] and converted into an object. The mouse genome was tiled in 1000 bp non-overlapping windows. Windows covered in all samples were retained and used for calling differentially CG/CH methylated regions with default parameters. DMRs were filtered to differences that were ≥ 5% between two groups with a SLIM-generated q-value less than 0.05. The methylBase and methylDiff objects were intersected to calculate percent methylation for each window passing filtering. Distribution of DMCGRs and DMCHRs within genic features and relative to the TSS was calculated using ChIPSeeker [127].

### DhMCGR analysis

To identify DhMCGRs, both BS and oxBS CpG text files were read into methylKit and converted into an object. 1000 bp non-overlapping windows covered in at least two samples were generated as above. The methylBase files generated for both BS and oxBS were read into methylKit and combined with percent methylation calculations to obtain a file containing percent methylation for each sample over windows passing filtering as described above and exported as a table. Percent methylation from BS and oxBS tiled regions was intersected using Bedtools, retaining only regions covered by both BS and oxBS. This intersected file was read back into RStudio and separated into two separate matrices containing BS and oxBS percent methylation. Hydroxymethylation over these regions was calculated by subtracting oxBS from BS for each region. DhMCGRs were filtered to differences ≥ 5% within each comparison (Astrocyte-Neuron, Microglia-Neuron, Astrocyte-Microglia), and assessment of the main effect of cell type was conducted using a Simple *T*-test (*p* ≤ 0.05) and manually calculated *q*-value ≤ 0.05 [*q* = min (*p*_i_ * *N*/rank_i_, q_i_ + 1)].

### Log odds over- and under-representation analysis

Log odds ratios were calculated for hyper- and hypo- DMCGRs, DhMCGRs, and DMCHRs within genic regions. methylBase files from each pairwise comparison containing all detected regions were used as the background. Using the R package ChIPseeker [127], the number of differentially modified regions within genic regions [Promoter (2-3 kb), Promoter (1-2 kb), Promoter (≥ 1 kb), 5′UTR, 1st Exon, 1st Intron, Other Exon, Other Intron, 3’UTR, Downstream (≤ 300 bp), and Distal Intergenic] were counted for hyper- and hypo-modifications from each comparison. Log odds ratios were calculated manually [ln((in comparison*out background)/(in background*out comparison))]. Genic regions not found to have any differentially modified regions within a given comparison were notated as “*n.d.*”. 95% confidence intervals and two-sided p-values were calculated using Woolf logit method and Fisher’s exact test, respectively.

### Targeted enzymatic methyl sequencing (EM-seq)

gDNA was isolated from the positive fraction of Camk2a-NuTRAP, Aldh1l1-NuTRAP, and Cx3cr1-NuTRAP mice (*n* = 3/group) via the INTACT protocol for targeted EM-seq of differentially hydroxymethylated regions identified from oxBS-seq. Two aliquots of 85 ng of DNA were prepared and brought up to 28 µL with H_2_O and taken through the enzymatic methyl conversion according to the manufacturer’s protocol, with slight modifications (Enzymatic Methyl-Seq Conversion Module, New England Biolabs, NEB #E7125). Briefly, one aliquot was taken through the enzymatic oxidation with TET2 and the other aliquot through a mock reaction supplementing an additional 4 µL of TET2 Reaction Buffer in place of TET2 enzyme. Oxidized or mock-converted DNA was cleaned using SPRIselect beads (1.75X) and eluted in 16 µL of Elution Buffer. DNA was denatured with 0.1 N sodium hydroxide and deamination of cytosines performed with APOBEC enzyme. Deaminated DNA was cleaned using SPRIselect beads (1X) and eluted in 30µL Elution Buffer. Primers were designed using the Methyl Primer Express v1.0 software (ThermoFisher Scientific) to amplify regions of the genome identified as having high differences in hydroxymethylation between cell types from oxBS-seq (Additional file 1C), and touchdown PCR was performed for a total of 40 cycles. Annealing temperatures were determined based on the Tm of each individual primer pair (only primers within Tm 1 °C of each other were run on the same plate). The initial annealing temperature (N_i_) was ~ 8–9 °C above the Tm, and the final annealing temperature (N_f_) was ~ 1–2 °C below the Tm. PCR conditions were as follows: [95 °C, 10 min; 10 (94 °C, 30 s; N_i_–N_f_ °C, 30 s; 72 °C, 30 s); 30 (94 °C, 30 s; N_f_ °C, 30 s; 72 °C, 30 s); 72 °C, 7 min; 4 °C, hold]. PCR amplified amplicons were cleaned using SPRIselect beads (1X) and eluted in 20 µL Elution Buffer. Clean amplicons were run on a 1% agarose gel to confirm size, and concentration of each amplicon (ng/µL) was determined using PicoGreen (Quant-iT PicoGreen dsDNA Assay Kit, ThermoFisher Scientific, Cat #P7589). A low-range standard curve (blank, 250 pg/mL, 2.5 ng/mL, and 25 ng/mL) was prepared. 25 ng of each amplicon were pooled together within a sample (individually for both TET + and TET- reactions), creating one pool for each sample and conversion group. Amplicon sizing was performed with HS D1000 screentape (#5067–5582; Agilent Technologies) and libraries were quantified using Qubit dsDNA HS Assay Kit (ThermoFisher Scientific). Pooled amplicons were diluted to 0.2 ng/µL with 1X low-EDTA TE buffer and taken through the Nextera XT library preparation (tagmentation, amplification, and clean-up) according to the manufacturer’s protocol (Nextera XT DNA Library Prep Kit, Illumina, #FC-131–1096; Nextera XT Index Kit v2, Illumina, #FC-131–2004). Libraries were quantified with Tapestation and Qubit, and 1 nM of each library was pooled together. Tapestation and Qubit was run again to verify library concentration. Libraries were diluted to a final concentration of 35 pM with Resuspension Buffer and PhiX control added for sequencing on the iSeq 100 (Illumina).

### EM-seq data analysis

Using CLC Genomics Workbench 20.0 (Qiagen), reads were first trimmed based on quality score of 0.05, a maximum of 2 ambiguities, trimming of Illumina universal adapters, removal of 8 5′ terminal nucleotides and 2 3′ nucleotides, and reads discarded below 75 bp and above 150 bp. The function “Map Bisulfite Reads to Reference” was used to align trimmed fastq files to bisulfite converted reference amplicon sequences in a non-directional fashion, no masking, with the following mapping options: match score of 1, mismatch cost of 2, linear gap cost with insertion and deletion cost of 3, length and similarity fraction of 0.8, mapping non-specific matches randomly. Methylation levels were called ignoring non-specific matches and broken pairs in the CpG, CHG & CHH context within a minimum strand-specific coverage of 10 and no statistical tests. CpG hydroxymethylation was determined using the methylation level of the mock non TET2-converted libraries. CpG methylation was determined by subtracting the methylation level of non TET2-converted libraries from TET2-converted libraries. Percent hmCG was plotted for the two cell types identified as having differential CG hydroxymethylation for each region, and CG methylation was plotted across these regions as well. A two-tailed *T*-test was performed on the average hmCG and mCG across each region, and a two-way ANOVA with Sidak’s multiple testing correction and single pooled variance was performed to assess hmCG and mCG at individual CpG sites.

### DNA modification and gene expression pattern analysis

Lists of high, mid, low, and not expressed genes were generated from raw RNA-seq transcript counts of Camk2a-NuTRAP, Aldh1l1-NuTRAP, and Cx3cr1-NuTRAP positive fraction samples. Genes with zero reads for all samples were classified as not expressed, with the remaining genes being split into three equally sized lists for high, mid, and low expressed genes. The R package EnrichedHeatmap was used to intersect methylation call files with genomic coordinates of gene lists according to expression level. The representative plots were generated and statistical analysis performed as described for oxBS-seq analysis.

### Software usage for analysis of transcriptomic and epigenomic data

DNA modification levels across genic regions were visualized using EnrichedHeatmap in R [123]. Distribution of DMRs within genic features and relative to the TSS [Promoter (2-3 kb), Promoter (1–2 kb), Promoter (≥ 1 kb), 5′UTR, 1st Exon, 1st Intron, Other Exon, Other Intron, 3′ UTR, Downstream (≤ 300 bp), and Distal Intergenic] were calculated using the R package ChIPseeker [127]. Transcription factor motif analysis was performed using Homer motif analysis software (v4.10) [61], and functional interpretations were compiled using the TFLink database (https://tflink.net/) [128] and cited literature.

### Supplementary Information

**Additional file 1: **Genotyping and RT-qPCR primers. Contains the genotyping primer sequences **A** and RT-qPCR TaqMan gene expression assays **B** used.

**Additional file 2: **Cell type-specific gene lists. Contains lists of neuronal, astrocytic, microglial, oligodendrocytic, and endothelial cell-specific genes

**Additional file 3: **Figure 2 additional information. Contains Pos vs Input fold change for each cell type specific gene (Additional file 2) from Camk2a-NuTRAP TRAP-isolated RNA-seq **A**, CIBERSORTx results **B**, Pos vs Input enriched genes **C**, Pos vs Input depleted genes **D**, GO biological processes of enriched genes **E**, IPA functions of enriched genes **F**, GO biological processes of depleted genes **G**, and IPA functions of depleted genes **H**.

**Additional file 4: **MiSeq CEGXQC reports. Contains CEGXQC reports from the initial MiSeq run performed to assess library quality and conversion efficiency. BS and oxBS reports for each sample are found under the same tab in the following order: 389Input, 389Neg, 389Pos, 391Input, 391Neg, 391Pos, 392Input, 392Neg, 392Pos, 396Input, 396Neg, 396Pos.

**Additional file 5: **NovaSeq 6000 FastQC reports. Contains FastQC reports from the NovaSeq 6000 run performed to obtain deeper sequencing on the Input and Positive fraction. BS and oxBS reports for each sample are found under the same tab in the following order: 389Input, 389Pos, 391Input, 391Pos, 392Input, 392Pos, 396Input, 396Pos.

**Additional file 6: **DMCGR motifs. Known HOMER motifs enriched in hyper Astrocyte vs Neuron **A**, hypo Astrocyte vs Neuron **B**, hyper Microglia vs Neuron **C**, hypo Microglia vs Neuron **D**, hyper Astrocyte vs Microglia **E**, and hypo Astrocyte vs Microglia DMCGRs.

**Additional file 7: **DhMCGR motifs. Known HOMER motifs enriched in hyper Astrocyte vs Neuron **A**, hypo Astrocyte vs Neuron **B**, hyper Microglia vs Neuron **C**, hypo Microglia vs Neuron **D**, hyper Astrocyte vs Microglia **E**, and hypo Astrocyte vs Microglia DhMCGRs.

**Additional file 8: **DMCHR motifs. Known HOMER motifs enriched in hyper Astrocyte vs Neuron **A**, hypo Astrocyte vs Neuron **B**, hyper Microglia vs Neuron **C**, hypo Microglia vs Neuron **D**, hyper Astrocyte vs Microglia **E**, and hypo Astrocyte vs Microglia DMCHRs

**Additional file 9: **Genes by expression level in neurons, astrocytes, and microglia. Contains genes by expression level (high, mid, low, and non-expressed) for neurons **A**, astrocytes **B**, and microglia **C**.

**Additional file 10: Figure S1.** Cre and Tamoxifen specificity of NuTRAP induction. Brains were harvested from Camk2a-cre+; NuTRAP+ (Camk2a-NuTRAP) mice, treated or not with tamoxifen (Tam), for immunohistochemical analysis of NuTRAP allele recombination or for assessment of neuronal, glial, and endothelial maker expression in the context of EGFP/mCherry localization. **A**–**B** Compared to counterparts from mice treated with Tam (+Tam), which exhibit robust efficiency of cre- neuronal recombination (nearly all neurons are positive for mCherry and EGFP), Camk2a-NuTRAP brains of mice not exposed to Tam (−Tam) display NuTRAP allele recombination to a subset of neurons (mCherry and EGFP expression localized to some NeuN+ cells). These data show a small degree of cre recombination specific to neurons independent of Tam induction (corroborating previously published observations) that is exacerbated by 5 days of systemic Tam delivery. **C** Camk2a-NuTRAP brains show no cre recombination (EGFP or mCherry expression) in cells expressing CD11b (microglia) **D** CD31 (endothelial), or **E** GFAP (astrocytes). DAPI: nuclei counterstain. Scale bar: 50 μm at 20X **A**, **B**, 50 μm at 40X **C**–**E**. **Figure S2.** Conversion efficiency of Camk2a-NuTRAP BS/oxBS-seq. **A** Summary of Bisulfite-sequencing (BS-Seq) and Oxidative Bisulfite-Sequencing (oxBS-Seq) techniques. Bisulfite-converted libraries are used to determine total percent modified cytosines (mC+hmC), while oxidative bisulfite-converted libraries are used to determine percent methylated cytosines (mC). hmC values are derived by subtracting oxBS from BS values on a per base basis. **B** Summary of Enzymatic Methyl-sequencing. TET-converted libraries (TET+) are used to determine total percent modified cytosines (mC+hmC), while non-TET-converted libraries (TET−) are used to determine percent hydroxymethylated cytosines (hmC). mC values are derived by subtracting TET- from TET+ values. **C**–**D**) Exogenous control sequences (CEGX, Cambridge, UK) were spiked in to each sheared DNA sample (0.04% *w*/*w*) prior to oxidation and/or bisulfite conversion. Raw fastq files were read into CEGXQC v0.2 to generate summary documentation and QC reports based on the conversion efficiency of the spike-in control sequences. Conversion percentages for different cytosine modifications (C, mC, and hmC) are plotted for bisulfite-converted **C** and oxidative bisulfite-converted **D** libraries. Bisulfite-converted libraries had near complete conversion of unmodified cytosines and low over-conversion of methylated and hydroxymethylated cytosines. Oxidative bisulfite-converted libraries had high levels of conversion of unmodified and hydroxymethylated cytosines and low conversion of methylated cytosines. *Note: one oxBS sample was missing the spike-in control so is not included in this plot*. **Figure S3.** DNA modifications across neuronal genes compared to all genes. mCG **A**, hmCG **B**, and mCH **C** averaged over 200 nucleotide bins from 4 kb upstream, within the gene body, and 4 kb downstream of neuronal marker genes (Additional file 2) and all genes from the positive fraction. **Figure S4.** Single cell RNA-seq expression of DNA modification regulators. Counts (Tabula Muris) or Normalized Counts (Allen Brain Atlas, Aging Mouse Brain) of DNA 58 modification regulators were plotted from single cell RNA-seq studies (one-way ANOVA with Tukey’s multiple comparisons test, *p < 0.05, **p < 0.01, ***p < 0.001, ****p < 0.0001). Additional file 1: **Table S1.** Average whole genome modification levels across detection methods for neurons, astrocytes, and microglia. Average whole genome mCG and hmCG levels were determined from oxBS-Seq and Nanopore data. The oxBS conversion correction was performed using conversion efficiency estimations based on CEGX spike-in control sequences and equations provided in Kozlenkov et al [3]. **Figure S5.** Repeat element modifications detected with nanopore sequencing. mCG **A** and hmCG **B** in specific repeat elements (LINE, SINE, LTR) were assessed from nanopore sequencing data for neurons, astrocytes, and microglia. One biological replicate is depicted for each cell type. **Figure S6.** Methylation across regions assessed with targeted EM-seq. Methylation of neurons, astrocytes, and microglia measured with targeted EM-seq (*n*=3/group) in six regions found to be differentially hydroxymethylated with WGoxBS. Line plots and total mCG across each region were plotted regions corresponding to *Chn1 ***A**, *Dlgap1* **B**, *Ankrd33b* **C**, *Dab2ip* **D**, *Chst2* **E**, and *Kalrn* **F **(two-way ANOVA with Sidak’s multiple testing correction and single pooled variance for individual CpG differences between cell types, two-tailed unpaired *t*-test for average region differences between cell types; *p<0.05, **p<0.01). **Figure S7.** Correlation of differential methylation and differential hydroxymethylation. mCG and hmCG differences in regions having both differential methylation and differential hydroxymethylation show a significant negative correlation with one another for **A** Astrocyte vs Neuron, **B** Microglia vs Neuron, and **C** Astrocyte vs Microglia comparisons (Simple linear regression with best fit line (solid), 95% confidence bands (dotted), and R^2^ goodness of fit). **Figure S8.** Overlap of gene lists by expression level between cell types. Venns of non-expressed **A**, low expressed **B**, mid expressed **C**, and high expressed **D** genes between neurons, astrocytes and microglia

## Data Availability

The WGoxBS datasets supporting the conclusions of this article are available in the GEO repository [Camk2a-NuTRAP oxBS-seq and RNA-seq: GSE228045; Aldh1l1-NuTRAP and Cx3cr1-NuTRAP oxBS-seq and RNA-seq: GSE140271, GSE140895, GSE159106] for download in FASTQ format. The Nanopore datasets are available in BioProject [PRJNA1026932] for download in FASTQ format. Other data that support the findings of the study are available from the corresponding author (W.M.F.) upon request.

## Abbreviations

CNS: Central nervous system
NuTRAP: Nuclear tagging and translating ribosome affinity purification
Tam: Tamoxifen
TRAP: Translating ribosome affinity purification
INTACT: Isolation of nuclei TAgged in specific cell types
WGoxBS: Whole genome oxidative bisulfite sequencing
BS-seq: Bisulfite sequencing
oxBS-seq: Oxidative bisulfite sequencing
DNMT: DNA methyltransferase
TET: Ten-eleven translocase
TDG: Thymine DNA glycosylase
LINE: Long interspersed nuclear element
SINE: Short interspersed nuclear element
LTR: Long terminal repeat
DMR: Differentially modified region
DMCGR: Differentially methylated CG region
DhMCGR: Differentially hydroxymethylated CG region
DMCHR: Differentially methylated CH region
EM-seq: Enzymatic methyl sequencing
