# Supplementary figures and images for: Differential usage of DNA modifications in neurons, astrocytes, and microglia
Source: Epigenetics Chromatin. 2023 Nov 13;16:45. doi: 10.1186/s13072-023-00522-6 (PMC10642035; doi:10.1186/s13072-023-00522-6)

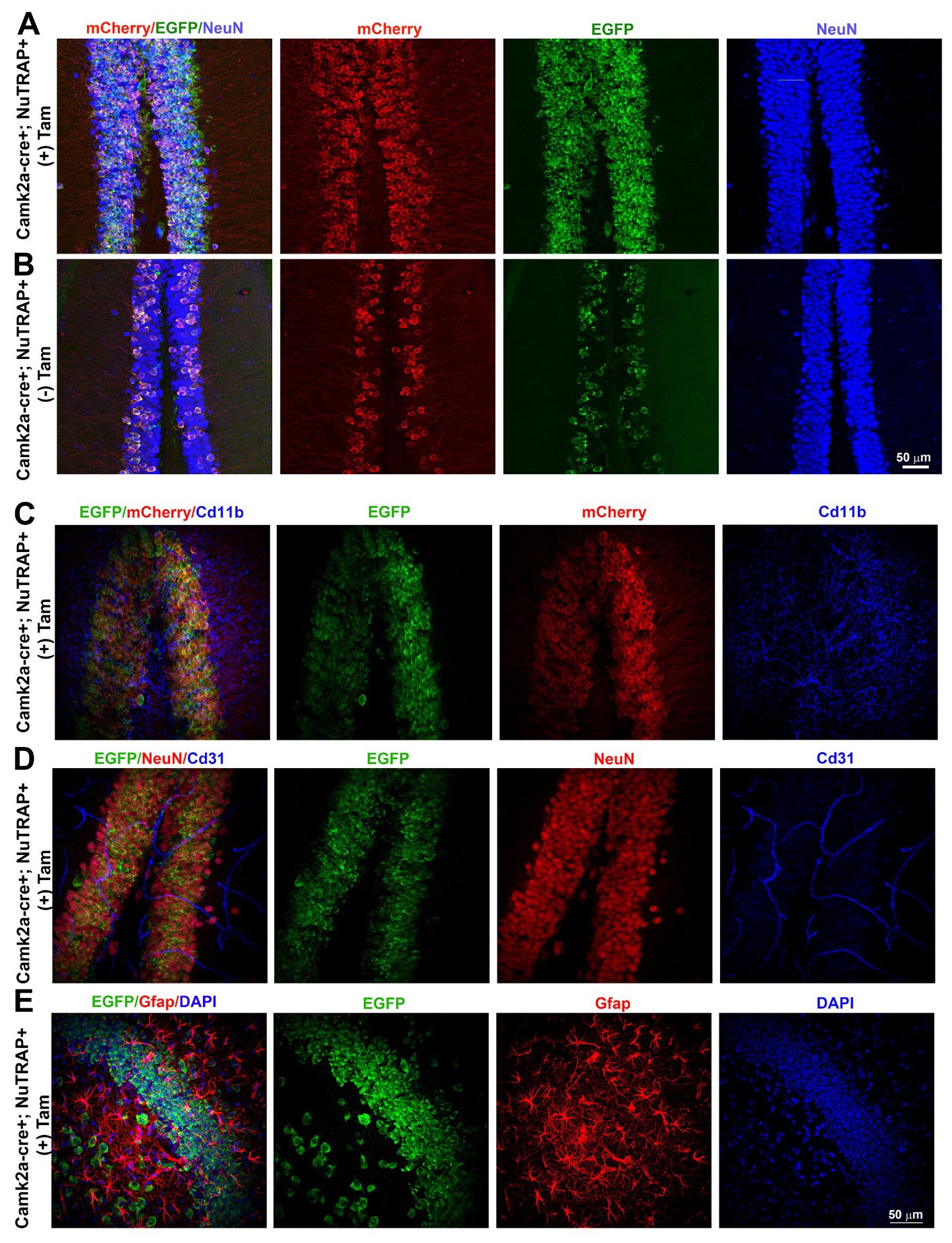

Supplement: Supplementary file 10 — Additional file 10: Figure S1. Cre and Tamoxifen specificity of NuTRAP induction. Brains were harvested from Camk2a-cre+; NuTRAP+ (Camk2a-NuTRAP) mice, treated or not with tamoxifen (Tam), for immunohistochemical analysis of NuTRAP allele recombination or for assessment of neuronal, glial, and endothelial maker expression in the context of EGFP/mCherry localization. A–B Compared to counterparts from mice treated with Tam (+Tam), which exhibit robust efficiency of cre- neuronal recombination (nearly all neurons are positive for mCherry and EGFP), Camk2a-NuTRAP brains of mice not exposed to Tam (−Tam) display NuTRAP allele recombination to a subset of neurons (mCherry and EGFP expression localized to some NeuN+ cells). These data show a small degree of cre recombination specific to neurons independent of Tam induction (corroborating previously published observations) that is exacerbated by 5 days of systemic Tam delivery. C Camk2a-NuTRAP brains show no cre recombination (EGFP or mCherry expression) in cells expressing CD11b (microglia) D CD31 (endothelial), or E GFAP (astrocytes). DAPI: nuclei counterstain. Scale bar: 50 μm at 20X A, B, 50 μm at 40X C–E. Figure S2. Conversion efficiency of Camk2a-NuTRAP BS/oxBS-seq. A Summary of Bisulfite-sequencing (BS-Seq) and Oxidative Bisulfite-Sequencing (oxBS-Seq) techniques. Bisulfite-converted libraries are used to determine total percent modified cytosines (mC+hmC), while oxidative bisulfite-converted libraries are used to determine percent methylated cytosines (mC). hmC values are derived by subtracting oxBS from BS values on a per base basis. B Summary of Enzymatic Methyl-sequencing. TET-converted libraries (TET+) are used to determine total percent modified cytosines (mC+hmC), while non-TET-converted libraries (TET−) are used to determine percent hydroxymethylated cytosines (hmC). mC values are derived by subtracting TET- from TET+ values. C–D) Exogenous control sequences (CEGX, Cambridge, UK) were spiked in to each sheare [file 13072_2023_522_MOESM10_ESM.zip › Additional file 10/Supplemental Figure 1.jpg]

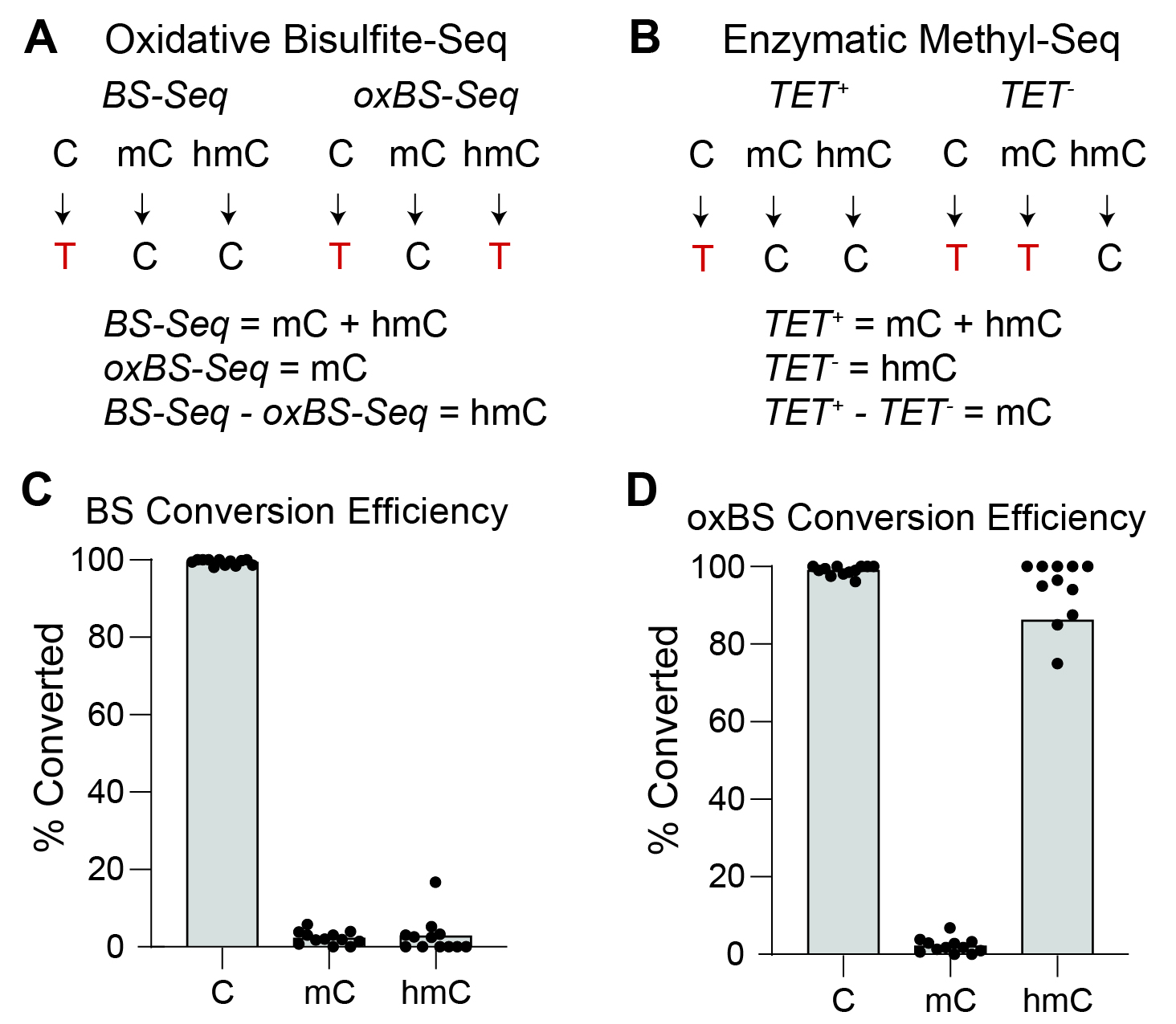

Supplement: Supplementary file 10 — Additional file 10: Figure S1. Cre and Tamoxifen specificity of NuTRAP induction. Brains were harvested from Camk2a-cre+; NuTRAP+ (Camk2a-NuTRAP) mice, treated or not with tamoxifen (Tam), for immunohistochemical analysis of NuTRAP allele recombination or for assessment of neuronal, glial, and endothelial maker expression in the context of EGFP/mCherry localization. A–B Compared to counterparts from mice treated with Tam (+Tam), which exhibit robust efficiency of cre- neuronal recombination (nearly all neurons are positive for mCherry and EGFP), Camk2a-NuTRAP brains of mice not exposed to Tam (−Tam) display NuTRAP allele recombination to a subset of neurons (mCherry and EGFP expression localized to some NeuN+ cells). These data show a small degree of cre recombination specific to neurons independent of Tam induction (corroborating previously published observations) that is exacerbated by 5 days of systemic Tam delivery. C Camk2a-NuTRAP brains show no cre recombination (EGFP or mCherry expression) in cells expressing CD11b (microglia) D CD31 (endothelial), or E GFAP (astrocytes). DAPI: nuclei counterstain. Scale bar: 50 μm at 20X A, B, 50 μm at 40X C–E. Figure S2. Conversion efficiency of Camk2a-NuTRAP BS/oxBS-seq. A Summary of Bisulfite-sequencing (BS-Seq) and Oxidative Bisulfite-Sequencing (oxBS-Seq) techniques. Bisulfite-converted libraries are used to determine total percent modified cytosines (mC+hmC), while oxidative bisulfite-converted libraries are used to determine percent methylated cytosines (mC). hmC values are derived by subtracting oxBS from BS values on a per base basis. B Summary of Enzymatic Methyl-sequencing. TET-converted libraries (TET+) are used to determine total percent modified cytosines (mC+hmC), while non-TET-converted libraries (TET−) are used to determine percent hydroxymethylated cytosines (hmC). mC values are derived by subtracting TET- from TET+ values. C–D) Exogenous control sequences (CEGX, Cambridge, UK) were spiked in to each sheare [file 13072_2023_522_MOESM10_ESM.zip › Additional file 10/Supplemental Figure 2.jpg]

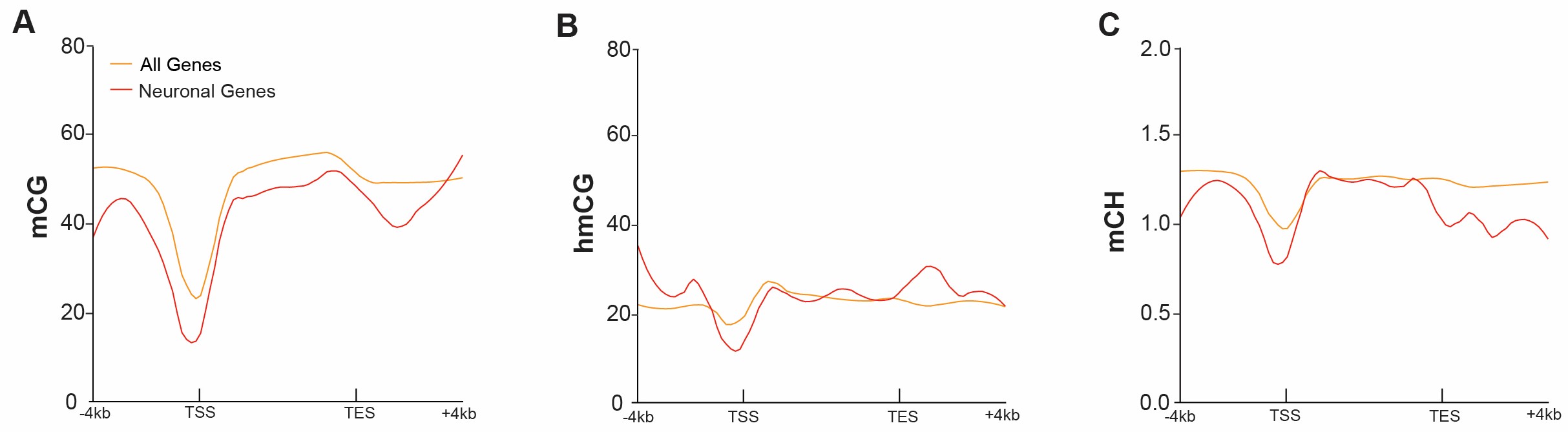

Supplement: Supplementary file 10 — Additional file 10: Figure S1. Cre and Tamoxifen specificity of NuTRAP induction. Brains were harvested from Camk2a-cre+; NuTRAP+ (Camk2a-NuTRAP) mice, treated or not with tamoxifen (Tam), for immunohistochemical analysis of NuTRAP allele recombination or for assessment of neuronal, glial, and endothelial maker expression in the context of EGFP/mCherry localization. A–B Compared to counterparts from mice treated with Tam (+Tam), which exhibit robust efficiency of cre- neuronal recombination (nearly all neurons are positive for mCherry and EGFP), Camk2a-NuTRAP brains of mice not exposed to Tam (−Tam) display NuTRAP allele recombination to a subset of neurons (mCherry and EGFP expression localized to some NeuN+ cells). These data show a small degree of cre recombination specific to neurons independent of Tam induction (corroborating previously published observations) that is exacerbated by 5 days of systemic Tam delivery. C Camk2a-NuTRAP brains show no cre recombination (EGFP or mCherry expression) in cells expressing CD11b (microglia) D CD31 (endothelial), or E GFAP (astrocytes). DAPI: nuclei counterstain. Scale bar: 50 μm at 20X A, B, 50 μm at 40X C–E. Figure S2. Conversion efficiency of Camk2a-NuTRAP BS/oxBS-seq. A Summary of Bisulfite-sequencing (BS-Seq) and Oxidative Bisulfite-Sequencing (oxBS-Seq) techniques. Bisulfite-converted libraries are used to determine total percent modified cytosines (mC+hmC), while oxidative bisulfite-converted libraries are used to determine percent methylated cytosines (mC). hmC values are derived by subtracting oxBS from BS values on a per base basis. B Summary of Enzymatic Methyl-sequencing. TET-converted libraries (TET+) are used to determine total percent modified cytosines (mC+hmC), while non-TET-converted libraries (TET−) are used to determine percent hydroxymethylated cytosines (hmC). mC values are derived by subtracting TET- from TET+ values. C–D) Exogenous control sequences (CEGX, Cambridge, UK) were spiked in to each sheare [file 13072_2023_522_MOESM10_ESM.zip › Additional file 10/Supplemental Figure 3.jpg]

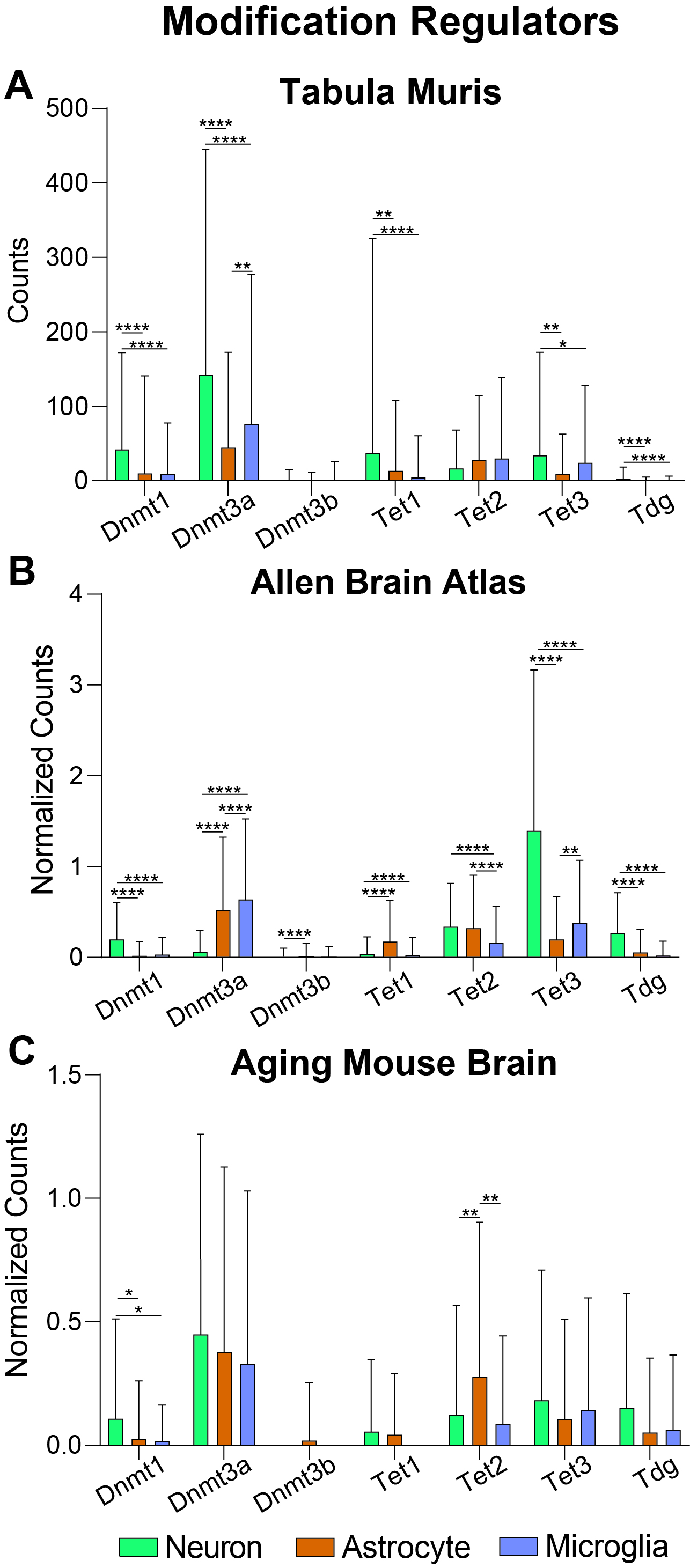

Supplement: Supplementary file 10 — Additional file 10: Figure S1. Cre and Tamoxifen specificity of NuTRAP induction. Brains were harvested from Camk2a-cre+; NuTRAP+ (Camk2a-NuTRAP) mice, treated or not with tamoxifen (Tam), for immunohistochemical analysis of NuTRAP allele recombination or for assessment of neuronal, glial, and endothelial maker expression in the context of EGFP/mCherry localization. A–B Compared to counterparts from mice treated with Tam (+Tam), which exhibit robust efficiency of cre- neuronal recombination (nearly all neurons are positive for mCherry and EGFP), Camk2a-NuTRAP brains of mice not exposed to Tam (−Tam) display NuTRAP allele recombination to a subset of neurons (mCherry and EGFP expression localized to some NeuN+ cells). These data show a small degree of cre recombination specific to neurons independent of Tam induction (corroborating previously published observations) that is exacerbated by 5 days of systemic Tam delivery. C Camk2a-NuTRAP brains show no cre recombination (EGFP or mCherry expression) in cells expressing CD11b (microglia) D CD31 (endothelial), or E GFAP (astrocytes). DAPI: nuclei counterstain. Scale bar: 50 μm at 20X A, B, 50 μm at 40X C–E. Figure S2. Conversion efficiency of Camk2a-NuTRAP BS/oxBS-seq. A Summary of Bisulfite-sequencing (BS-Seq) and Oxidative Bisulfite-Sequencing (oxBS-Seq) techniques. Bisulfite-converted libraries are used to determine total percent modified cytosines (mC+hmC), while oxidative bisulfite-converted libraries are used to determine percent methylated cytosines (mC). hmC values are derived by subtracting oxBS from BS values on a per base basis. B Summary of Enzymatic Methyl-sequencing. TET-converted libraries (TET+) are used to determine total percent modified cytosines (mC+hmC), while non-TET-converted libraries (TET−) are used to determine percent hydroxymethylated cytosines (hmC). mC values are derived by subtracting TET- from TET+ values. C–D) Exogenous control sequences (CEGX, Cambridge, UK) were spiked in to each sheare [file 13072_2023_522_MOESM10_ESM.zip › Additional file 10/Supplemental Figure 4.jpg]

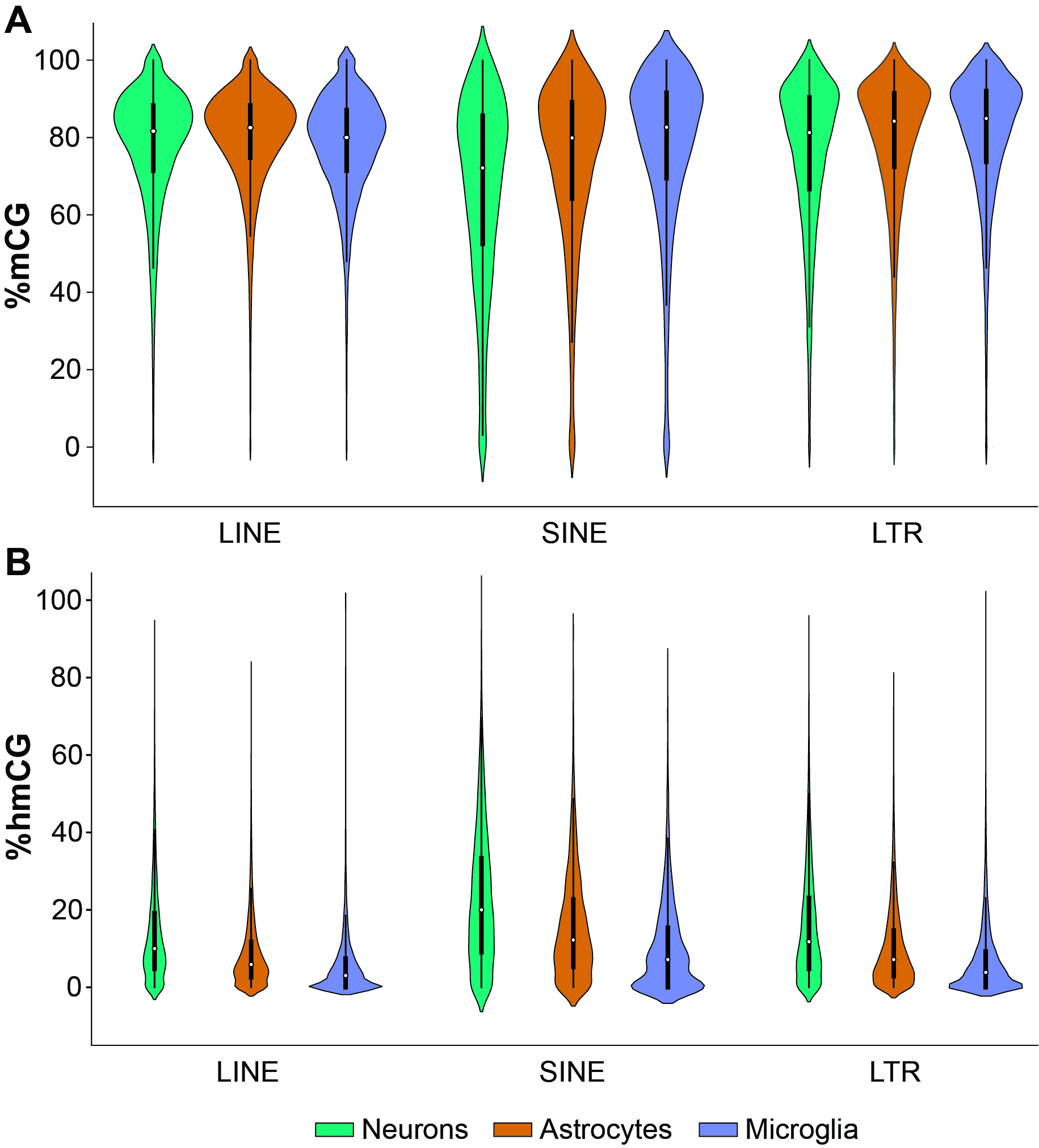

Supplement: Supplementary file 10 — Additional file 10: Figure S1. Cre and Tamoxifen specificity of NuTRAP induction. Brains were harvested from Camk2a-cre+; NuTRAP+ (Camk2a-NuTRAP) mice, treated or not with tamoxifen (Tam), for immunohistochemical analysis of NuTRAP allele recombination or for assessment of neuronal, glial, and endothelial maker expression in the context of EGFP/mCherry localization. A–B Compared to counterparts from mice treated with Tam (+Tam), which exhibit robust efficiency of cre- neuronal recombination (nearly all neurons are positive for mCherry and EGFP), Camk2a-NuTRAP brains of mice not exposed to Tam (−Tam) display NuTRAP allele recombination to a subset of neurons (mCherry and EGFP expression localized to some NeuN+ cells). These data show a small degree of cre recombination specific to neurons independent of Tam induction (corroborating previously published observations) that is exacerbated by 5 days of systemic Tam delivery. C Camk2a-NuTRAP brains show no cre recombination (EGFP or mCherry expression) in cells expressing CD11b (microglia) D CD31 (endothelial), or E GFAP (astrocytes). DAPI: nuclei counterstain. Scale bar: 50 μm at 20X A, B, 50 μm at 40X C–E. Figure S2. Conversion efficiency of Camk2a-NuTRAP BS/oxBS-seq. A Summary of Bisulfite-sequencing (BS-Seq) and Oxidative Bisulfite-Sequencing (oxBS-Seq) techniques. Bisulfite-converted libraries are used to determine total percent modified cytosines (mC+hmC), while oxidative bisulfite-converted libraries are used to determine percent methylated cytosines (mC). hmC values are derived by subtracting oxBS from BS values on a per base basis. B Summary of Enzymatic Methyl-sequencing. TET-converted libraries (TET+) are used to determine total percent modified cytosines (mC+hmC), while non-TET-converted libraries (TET−) are used to determine percent hydroxymethylated cytosines (hmC). mC values are derived by subtracting TET- from TET+ values. C–D) Exogenous control sequences (CEGX, Cambridge, UK) were spiked in to each sheare [file 13072_2023_522_MOESM10_ESM.zip › Additional file 10/Supplemental Figure 5.jpg]

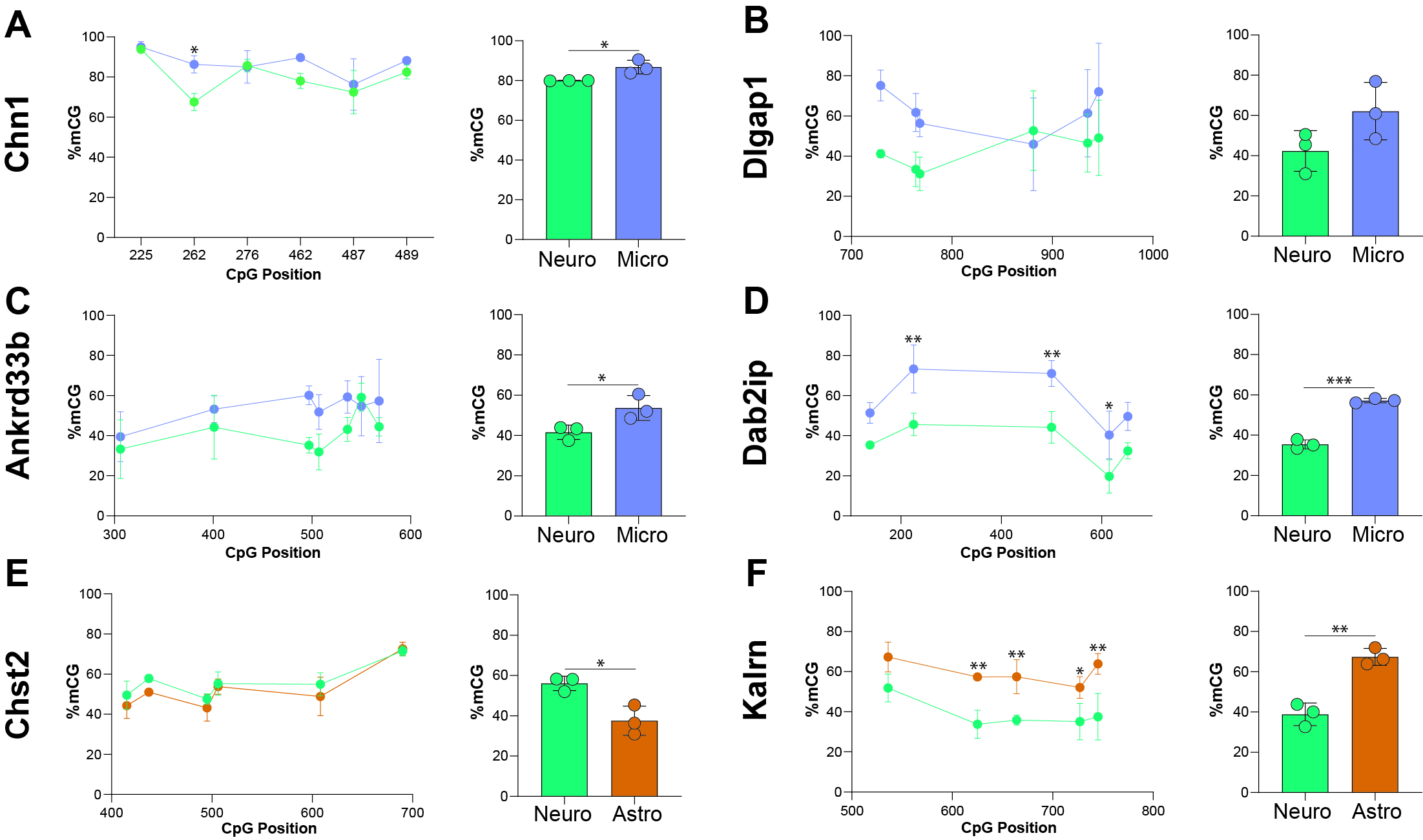

Supplement: Supplementary file 10 — Additional file 10: Figure S1. Cre and Tamoxifen specificity of NuTRAP induction. Brains were harvested from Camk2a-cre+; NuTRAP+ (Camk2a-NuTRAP) mice, treated or not with tamoxifen (Tam), for immunohistochemical analysis of NuTRAP allele recombination or for assessment of neuronal, glial, and endothelial maker expression in the context of EGFP/mCherry localization. A–B Compared to counterparts from mice treated with Tam (+Tam), which exhibit robust efficiency of cre- neuronal recombination (nearly all neurons are positive for mCherry and EGFP), Camk2a-NuTRAP brains of mice not exposed to Tam (−Tam) display NuTRAP allele recombination to a subset of neurons (mCherry and EGFP expression localized to some NeuN+ cells). These data show a small degree of cre recombination specific to neurons independent of Tam induction (corroborating previously published observations) that is exacerbated by 5 days of systemic Tam delivery. C Camk2a-NuTRAP brains show no cre recombination (EGFP or mCherry expression) in cells expressing CD11b (microglia) D CD31 (endothelial), or E GFAP (astrocytes). DAPI: nuclei counterstain. Scale bar: 50 μm at 20X A, B, 50 μm at 40X C–E. Figure S2. Conversion efficiency of Camk2a-NuTRAP BS/oxBS-seq. A Summary of Bisulfite-sequencing (BS-Seq) and Oxidative Bisulfite-Sequencing (oxBS-Seq) techniques. Bisulfite-converted libraries are used to determine total percent modified cytosines (mC+hmC), while oxidative bisulfite-converted libraries are used to determine percent methylated cytosines (mC). hmC values are derived by subtracting oxBS from BS values on a per base basis. B Summary of Enzymatic Methyl-sequencing. TET-converted libraries (TET+) are used to determine total percent modified cytosines (mC+hmC), while non-TET-converted libraries (TET−) are used to determine percent hydroxymethylated cytosines (hmC). mC values are derived by subtracting TET- from TET+ values. C–D) Exogenous control sequences (CEGX, Cambridge, UK) were spiked in to each sheare [file 13072_2023_522_MOESM10_ESM.zip › Additional file 10/Supplemental Figure 6.jpg]

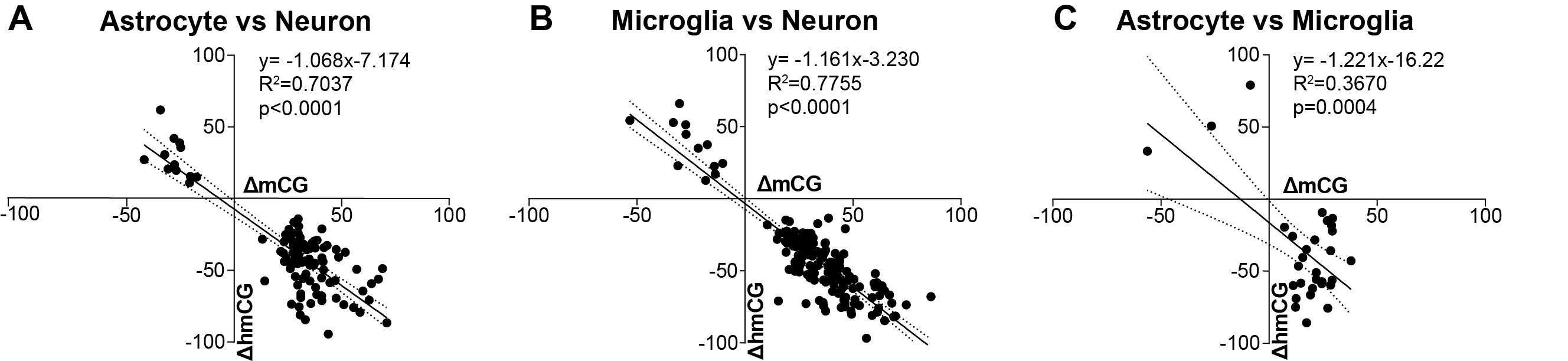

Supplement: Supplementary file 10 — Additional file 10: Figure S1. Cre and Tamoxifen specificity of NuTRAP induction. Brains were harvested from Camk2a-cre+; NuTRAP+ (Camk2a-NuTRAP) mice, treated or not with tamoxifen (Tam), for immunohistochemical analysis of NuTRAP allele recombination or for assessment of neuronal, glial, and endothelial maker expression in the context of EGFP/mCherry localization. A–B Compared to counterparts from mice treated with Tam (+Tam), which exhibit robust efficiency of cre- neuronal recombination (nearly all neurons are positive for mCherry and EGFP), Camk2a-NuTRAP brains of mice not exposed to Tam (−Tam) display NuTRAP allele recombination to a subset of neurons (mCherry and EGFP expression localized to some NeuN+ cells). These data show a small degree of cre recombination specific to neurons independent of Tam induction (corroborating previously published observations) that is exacerbated by 5 days of systemic Tam delivery. C Camk2a-NuTRAP brains show no cre recombination (EGFP or mCherry expression) in cells expressing CD11b (microglia) D CD31 (endothelial), or E GFAP (astrocytes). DAPI: nuclei counterstain. Scale bar: 50 μm at 20X A, B, 50 μm at 40X C–E. Figure S2. Conversion efficiency of Camk2a-NuTRAP BS/oxBS-seq. A Summary of Bisulfite-sequencing (BS-Seq) and Oxidative Bisulfite-Sequencing (oxBS-Seq) techniques. Bisulfite-converted libraries are used to determine total percent modified cytosines (mC+hmC), while oxidative bisulfite-converted libraries are used to determine percent methylated cytosines (mC). hmC values are derived by subtracting oxBS from BS values on a per base basis. B Summary of Enzymatic Methyl-sequencing. TET-converted libraries (TET+) are used to determine total percent modified cytosines (mC+hmC), while non-TET-converted libraries (TET−) are used to determine percent hydroxymethylated cytosines (hmC). mC values are derived by subtracting TET- from TET+ values. C–D) Exogenous control sequences (CEGX, Cambridge, UK) were spiked in to each sheare [file 13072_2023_522_MOESM10_ESM.zip › Additional file 10/Supplemental Figure 7.jpg]

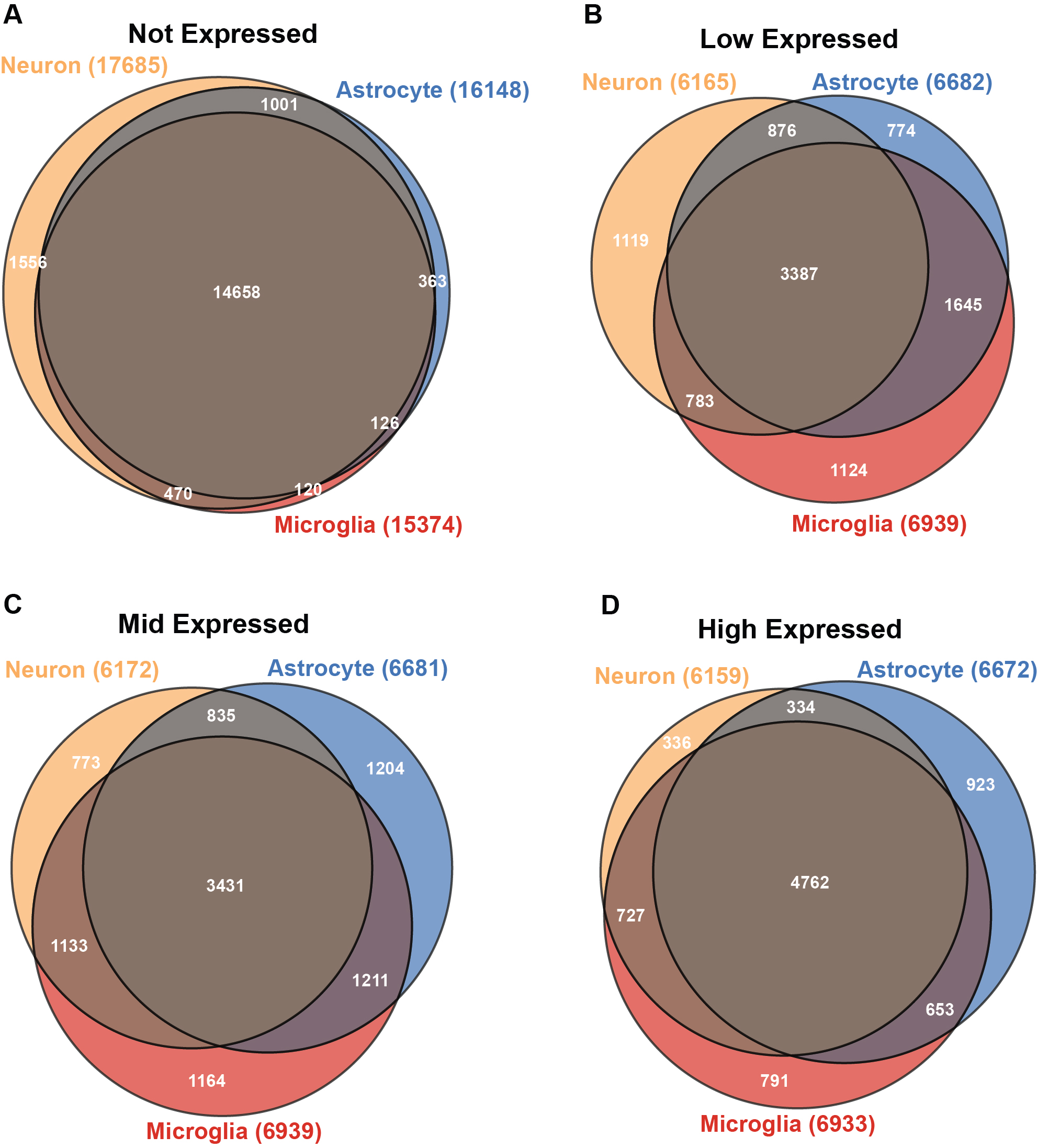

Supplement: Supplementary file 10 — Additional file 10: Figure S1. Cre and Tamoxifen specificity of NuTRAP induction. Brains were harvested from Camk2a-cre+; NuTRAP+ (Camk2a-NuTRAP) mice, treated or not with tamoxifen (Tam), for immunohistochemical analysis of NuTRAP allele recombination or for assessment of neuronal, glial, and endothelial maker expression in the context of EGFP/mCherry localization. A–B Compared to counterparts from mice treated with Tam (+Tam), which exhibit robust efficiency of cre- neuronal recombination (nearly all neurons are positive for mCherry and EGFP), Camk2a-NuTRAP brains of mice not exposed to Tam (−Tam) display NuTRAP allele recombination to a subset of neurons (mCherry and EGFP expression localized to some NeuN+ cells). These data show a small degree of cre recombination specific to neurons independent of Tam induction (corroborating previously published observations) that is exacerbated by 5 days of systemic Tam delivery. C Camk2a-NuTRAP brains show no cre recombination (EGFP or mCherry expression) in cells expressing CD11b (microglia) D CD31 (endothelial), or E GFAP (astrocytes). DAPI: nuclei counterstain. Scale bar: 50 μm at 20X A, B, 50 μm at 40X C–E. Figure S2. Conversion efficiency of Camk2a-NuTRAP BS/oxBS-seq. A Summary of Bisulfite-sequencing (BS-Seq) and Oxidative Bisulfite-Sequencing (oxBS-Seq) techniques. Bisulfite-converted libraries are used to determine total percent modified cytosines (mC+hmC), while oxidative bisulfite-converted libraries are used to determine percent methylated cytosines (mC). hmC values are derived by subtracting oxBS from BS values on a per base basis. B Summary of Enzymatic Methyl-sequencing. TET-converted libraries (TET+) are used to determine total percent modified cytosines (mC+hmC), while non-TET-converted libraries (TET−) are used to determine percent hydroxymethylated cytosines (hmC). mC values are derived by subtracting TET- from TET+ values. C–D) Exogenous control sequences (CEGX, Cambridge, UK) were spiked in to each sheare [file 13072_2023_522_MOESM10_ESM.zip › Additional file 10/Supplemental Figure 8.jpg]

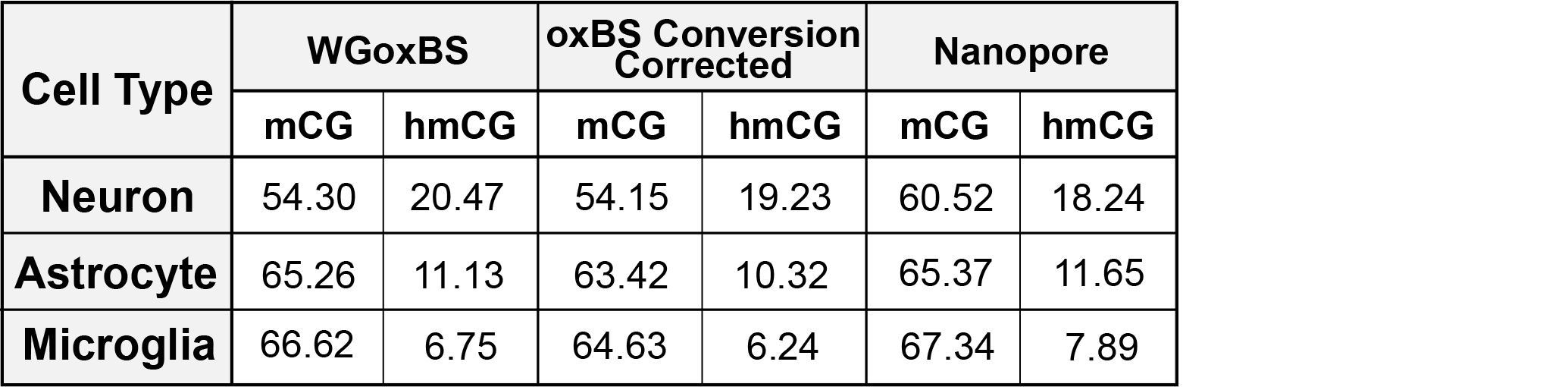

Supplement: Supplementary file 10 — Additional file 10: Figure S1. Cre and Tamoxifen specificity of NuTRAP induction. Brains were harvested from Camk2a-cre+; NuTRAP+ (Camk2a-NuTRAP) mice, treated or not with tamoxifen (Tam), for immunohistochemical analysis of NuTRAP allele recombination or for assessment of neuronal, glial, and endothelial maker expression in the context of EGFP/mCherry localization. A–B Compared to counterparts from mice treated with Tam (+Tam), which exhibit robust efficiency of cre- neuronal recombination (nearly all neurons are positive for mCherry and EGFP), Camk2a-NuTRAP brains of mice not exposed to Tam (−Tam) display NuTRAP allele recombination to a subset of neurons (mCherry and EGFP expression localized to some NeuN+ cells). These data show a small degree of cre recombination specific to neurons independent of Tam induction (corroborating previously published observations) that is exacerbated by 5 days of systemic Tam delivery. C Camk2a-NuTRAP brains show no cre recombination (EGFP or mCherry expression) in cells expressing CD11b (microglia) D CD31 (endothelial), or E GFAP (astrocytes). DAPI: nuclei counterstain. Scale bar: 50 μm at 20X A, B, 50 μm at 40X C–E. Figure S2. Conversion efficiency of Camk2a-NuTRAP BS/oxBS-seq. A Summary of Bisulfite-sequencing (BS-Seq) and Oxidative Bisulfite-Sequencing (oxBS-Seq) techniques. Bisulfite-converted libraries are used to determine total percent modified cytosines (mC+hmC), while oxidative bisulfite-converted libraries are used to determine percent methylated cytosines (mC). hmC values are derived by subtracting oxBS from BS values on a per base basis. B Summary of Enzymatic Methyl-sequencing. TET-converted libraries (TET+) are used to determine total percent modified cytosines (mC+hmC), while non-TET-converted libraries (TET−) are used to determine percent hydroxymethylated cytosines (hmC). mC values are derived by subtracting TET- from TET+ values. C–D) Exogenous control sequences (CEGX, Cambridge, UK) were spiked in to each sheare [file 13072_2023_522_MOESM10_ESM.zip › Additional file 10/Supplemental Table 1.jpg]
